# Pathway reprogramming and catalytic network engineering for the production of bioactive aspertetranones from deep-sea *Aspergillus versicolor* ADS-F20

**DOI:** 10.1016/j.engmic.2025.100259

**Published:** 2026-01-02

**Authors:** Peiyuan Feng, Moli Sang, Wei Zhang

**Affiliations:** a Laboratory of Experimental Marine Biology, Institute of Oceanology, Chinese Academy of Sciences, Qingdao 266000, China; b State Key Laboratory of Microbial Technology, Shandong University, Qingdao 266237, China; c Laboratory for Marine Biology and Biotechnology, Qingdao Marine Science and Technology Center, Qingdao 266237, China; d Shenzhen Research Institute of Shandong University, Shenzhen 518057, China

**Keywords:** Fungal meroterpenoids, Aspertetranones, Combinatorial biosynthesis, Pathway reprogramming, Bioactivity

## Abstract

Aspertetranones are a unique class of marine fungal meroditerpenoids characterized by a highly oxygenated, linear 6/6/6/6 tetracyclic core fused to an α-pyrone scaffold. Although the pathway of aspertetranone biosynthesis in *Aspergillus ochraceopetaliformis* has been partially elucidated, the full potential of these compounds remains untapped. The structural diversity and enzyme promiscuity of tailoring reactions offer unexplored opportunities for the generation of bioactive derivatives through combinatorial biosynthesis. In this study, we identified the *atn* biosynthetic gene cluster responsible for aspertetranone production in deep-sea-derived *Aspergillus versicolor* ADS-F20. Through the systematic heterologous expression of 12 key genes in *Aspergillus oryzae*, the full pathway reconstitution and targeted biosynthesis of 17 metabolites were achieved, thus expanding the known chemical space of meroterpenoids. Notably, bioactivity screening identified compound **6** as having potent antibacterial and antifungal activities against *Vibrio vulnificus* ATCC 27562 (MIC = 4.50 μg/mL) and *Phytophthora nicotianae* (MIC = 9.01 μg/mL). Compound **11** demonstrated broad-spectrum anticancer and cytotoxic effects against the K-562, MCF7, and PATU8988T cell lines. This study underscores the power of pathway reprogramming and catalytic network engineering as versatile strategies for expanding the structural and functional diversity of biosynthetic pathway components.

## Graphical abstract

**Figure:**
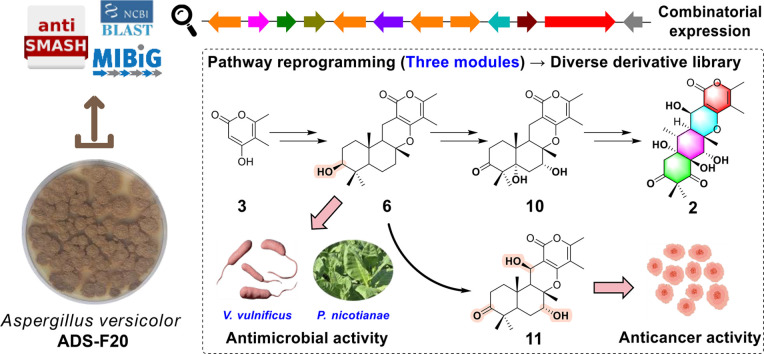

## 1 Introduction

**Fig. 1:**
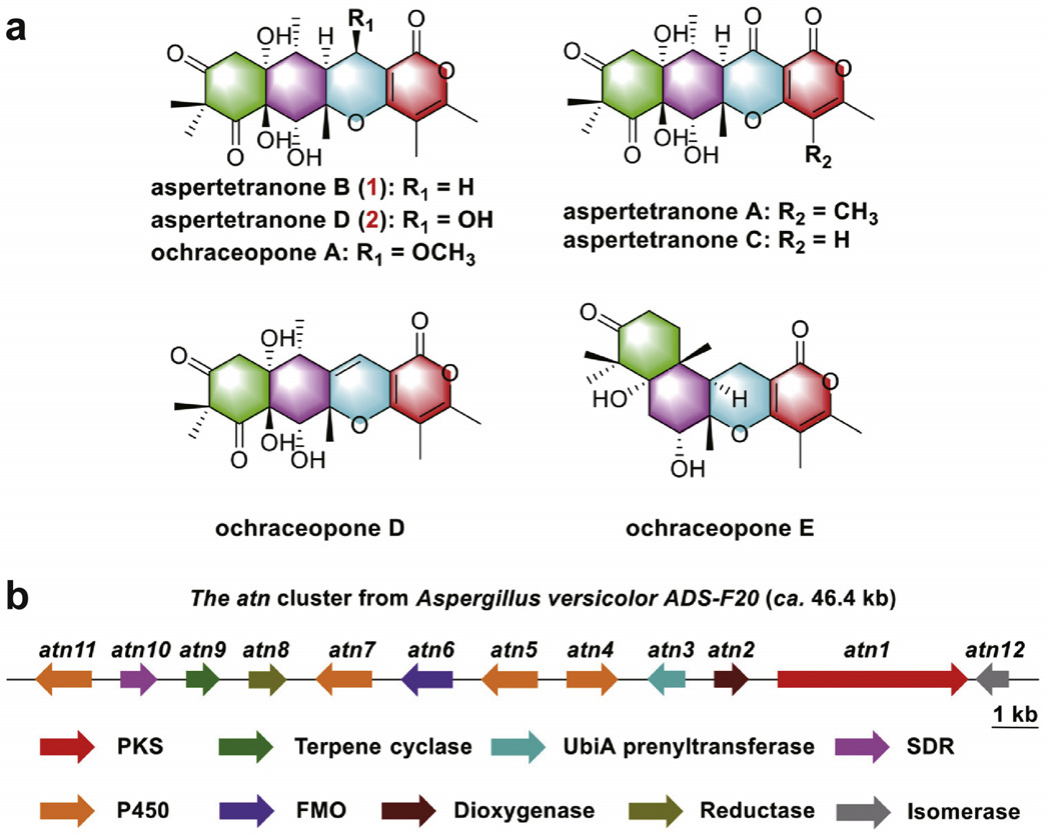
(a) Chemical structures of the aspertetranone family. (b) Putative gene cluster *atn* from *Aspergillus versicolor* ADS-F20. PKS: polyketide synthase; FMO: flavin-dependent monooxygenase; SDR: short-chain dehydrogenase/reductase; P450: cytochrome P450.

Fungal meroterpenoids represent a structurally diverse class of hybrid natural products that incorporate biosynthetic units from terpenoid and polyketide pathways [[1], [2], [3]]. Their complex chemical structures and broad spectrum of bioactivities have made them important scaffolds for drug discovery [4]. Among these natural products, aspertetranones from marine fungi were the first triketide-sesquiterpenoids reported, featuring an unprecedented linear 6/6/6/6 tetracyclic core (Fig. 1a). They were first isolated from the marine algal-associated fungus *Aspergillus* sp. ZL0-1b14 [5] and later from Antarctic soil-derived *Aspergillus ochraceopetaliformis* SCSIO 05702 [6], and were highlighted as “hot-spot” structures in *Natural Product Reports* [7]. Biological evaluations indicated that aspertetranones A and D at 40 μM concentration exhibited anti-inflammatory activity (43 ± 2% and 69 ± 2% against interleukin-6 and 42 ± 2% and 47 ± 4% against interleukin-1 beta, respectively). Additionally, the analog ochraceopone A was found to have obvious antiviral activity toward the H1N1 and H3N2 influenza viruses, with IC_50_ values of more than 20.0 ± 4.10 and 12.2 ± 4.10 μM, respectively.

In 2024, Yan et al. and Lin et al. independently deciphered the biosynthetic pathways of ochraceopones and aspertetranones by identifying and characterizing their respective biosynthetic gene clusters (BGCs) *och* and *ato* [8,9]. The biosynthesis of aspertetranones is a complex modular process wherein the characteristic linear tetracyclic scaffold is derived from an angular tetracyclic precursor and subsequently elaborated by a multi-enzyme cascade. However, it remains unclear whether the combinatorial expression of diverse post-modification enzymes can generate more derivatives and facilitate one-step directed synthesis and whether biosynthetic angular tetracyclic intermediates with better biological activity exist.

Recently, we identified aspertetranone derivatives from *Aspergillus versicolor* ADS-F20, a deep-sea Arctic mud-derived fungus (**Fig. S1**). Through genome mining, we identified the BGC *atn* and heterologously reconstituted the full pathway in *Aspergillus oryzae*, thereby establishing a robust microbial platform for scalable aspertetranone production. Leveraging the remarkable substrate promiscuity of tailoring oxidoreductases, including Atn2, Atn5, Atn7, Atn10, and Atn11, we rationally engineered this system to generate a structurally diverse derivative library. This combinatorial biosynthesis strategy not only demonstrated the flexibility of the aspertetranone pathway but also yielded a rich chemical repertoire for biological evaluation. Subsequent screening of the isolated compounds for anti-inflammatory, antimicrobial, and anticancer activities identified a few promising candidates. These findings provide important insights into the structure–activity relationships of aspertetranone scaffolds and a foundation for rational drug design and therapeutic development.

## 2 Materials and methods

### 2.1 General experimental procedures

Unless otherwise specified, all chemicals and antibiotics used in this study were obtained from Aladdin (Shanghai, China), Solarbio (Beijing, China), and Sinopharm Chemical Reagent Co., Ltd. (Shanghai, China). HPLC-grade solvents were purchased from Merck KGaA Ltd. (Darmstadt, Germany), and analytical organic solvents for compound isolation and purification were procured from Fuyu Fine Chemical Co., Ltd. (Tianjin, China). Sephadex® LH-20 was purchased from Cytiva (Danaher Corporation, Washington, DC, USA). Both analytical and semi-preparative HPLC were performed on a Thermo Scientific™ Vanquish™ UHPLC system (Dionex Softron GmbH, Germering, Germany). LC-HRMS analysis was performed using a Bruker Impact HD High-Resolution Q-TOF mass spectrometer. NMR spectra (^1^H, ^13^C, ^1^H-^1^H COSY, HSQC, and HMBC) were obtained with a Bruker Avance III 600 HD (Cryo) MHz spectrometer. All restriction enzymes were purchased from New England Biolabs, Inc. (Ipswich, MA, USA) and Thermo Fisher Scientific (Pittsburgh, PA, USA). The High-Fidelity DNA Polymerase 2 × Phanta Max Master Mix from Vazyme Biotech (Nanjing, China) was used to amplify DNA fragments, whereas the 2 × T5 Super PCR Mix from Tsingke (Beijing, China) was used for colony PCR identification. The designing of the oligonucleotide primers and DNA sequencing were performed by Sangon Biotech (Shanghai, China). The ClonExpress Ultra One Step Cloning Kit (Vazyme Biotech) was used for plasmid construction. The E.Z.N.A.^TM^ Plasmid Miniprep Kit (Omega Biotek, Norcross, GA, USA) and E.Z.N.A.^TM^ Gel Extraction Kit (Omega Biotek) were used for plasmid extraction and DNA fragment purification, respectively. The Ni-NTA Sefinose^TM^ Resin (Settled Resin) for protein purification was acquired from Sangon Biotech. The FlexiRun premixed gel solution for SDS-PAGE was obtained from MD Bio (Qingdao, China). All 19 test strains were purchased from the China General Microbiological Culture Collection Center and China Center of Industrial Culture Collection.

### 2.2 Strains and media

The aspertetranone-producing marine-derived fungus *A. versicolor* ADS-F20 was cultivated at 30°C for 10 days on PDA medium (Becton Dickinson and Company, Franklin Lakes, NJ, USA) supplemented with 3.3% sea salt. The strain was used for genomic DNA extraction and as the source for the cloning of each gene of the *atn* BGC. The quadruple auxotrophic *A. oryzae* NSAR1 (*niaD^-^, sC^-^, ΔargB, adeA^-^*) [10] was used as the heterologous host for fungal gene expression. The *A. oryzae* transformants were grown in shaking cultures in CMP medium (3.5% Czapek–Dox broth, 3% maltose, and 1% peptone) for 3–5 days at 30°C and 200 rpm to produce the metabolites derived from the introduced genes. Standard DNA engineering experiments (vector construction and plasmid preparation) were performed using *Escherichia coli* DH5α (Tsingke Biotechnology Co., Ltd). The *E. coli* BL21 (DE3) strain was used to express the His_6_-tagged proteins Atn2 and Atn10. *E. coli* cells carrying each plasmid were cultivated in Luria-Bertani (LB) medium and selected using 100 mg/L ampicillin or 50 mg/L kanamycin sulfate.

### 2.3 Whole-genome sequencing and bioinformatic analysis

Whole-genome sequencing of *A. versicolor* ADS-F20 was performed by Biomarker Technologies Co., Ltd. (Beijing, China) using an Illumina HiSeq system. The resulting short reads were assembled using SPAdes version 3.6.2, yielding 302 scaffolds covering approximately 2.51 Mb. AntiSMASH 7.0 [11] was used to identify, annotate, and analyze gene clusters associated with the biosynthesis of secondary metabolites, and NCBI-BLAST (https://blast.ncbi.nlm.nih.gov/Blast.cgi) and FGENESH (http://www.softberry.com) were further used for gene prediction, with sequences manually revised if necessary.

### 2.4 Construction of expression plasmids for fungal transformation

Each gene in the *atn* BGC was amplified from *A. versicolor* ADS-F20 genomic DNA using the primers listed in **Table S1**. The full-length genes (*atn2*–*atn12*) were purified and ligated into the pTAex3 vector [12], whereas *atn1* was ligated into the pUSA vector [13], using the Clonexpress® Ultra One Step Cloning Kit (Vazyme Biotech Co., Ltd) according to the manufacturer’s protocol. To introduce the relevant genes (*atn2, atn5*, and *atn10*) into pAdeA [14], the targeted DNA fragment containing the amyB promoter (PamyB) and amyB terminator (TamyB) was amplified from the above-described constructed pTAex3-based plasmids and ligated into the *sbf*I-digested promoter/terminator-free vector. For multigene-containing vector construction, gene-of-interest fragments containing both PamyB and TamyB were also amplified from the pTAex3-based plasmids and ligated into each vector, as detailed in **Table S2**. All constructed plasmids were verified by DNA sequencing.

### 2.5 Transformation of Aspergillus oryzae NSAR1

Transformation of *A. oryzae* NSAR1 and its mutants was performed using a previously described protoplast-polyethylene glycol method [15]. The spore suspension of *A. oryzae* NSAR1 or the mutants was inoculated into 100 mL of DPY liquid medium (2% dextrin, 1% polypeptone, 0.5% yeast extract, 0.5% KH_2_PO_4_, and 0.05% MgSO_4_•7H_2_O) and incubated for 36 h at 30°C and 200 rpm. Mycelia were collected by filter paper filtration and then used for the preparation of protoplasts by soaking in an 0.8 M NaCl solution containing 10% lywallzyme, 10% snailase, 10% cellulase, and 10% lysozyme with gentle shaking at 30°C for 2–3 h. The resultant protoplast-containing mixture was filtered, and the filtrate was centrifuged at 4500 rpm for 5 min and washed using Solution I (0.8 M NaCl, 10 mM CaCl_2_, and 50 mM Tris-HCl, pH 7.5). The protoplasts were resuspended in Solution I to a final concentration of 3 × 10^7^ cells/mL. A mixture of 120 μL protoplast solution and 5–10 µg transformation plasmids (<10 μL) was incubated on ice for 2 min. Subsequently, 1 mL of Solution II (60% (w/v) PEG 6000, 0.8 M NaCl, 50 mM CaCl_2_, and 50 mM Tris-HCl, pH 7.5) was added, and the mixture was further incubated for 20 min at 25°C. After pre-insulation, 7 mL of CDS soft agar (3.5% Czapek-Dox broth, 1 M sorbitol, and 0.7% agar) was added, and the entire mixture was then poured on top of CDS medium plates (3.5% Czapek-Dox broth, 1 M sorbitol, and 1.5% agar). The plates were incubated at 30°C for 3–6 days until transformants appeared. All the transformants constructed in this study are listed in **Table S3**.

### 2.6 HPLC, UPLC, and LC-HRMS analyses of each product

The products from each of the *A. oryzae* transformants were analyzed using HPLC, whereas those from *in vitro* enzymatic reactions were analyzed using UPLC. All HPLC analyses were carried out on a YMC-Triart C_18_ column (250 mm × 4.6 mm, 5 μm, UV monitored at 280 nm) with a biphasic solvent system consisting of solvent B (ACN) and solvent A (H_2_O + 0.1% TFA) flowing at a rate of 1.0 mL/min. The column temperature was set at 25°C, and the program was as follows: 0–3 min, 10%; 3–23 min, 10–100%; 23–31 min, 100%; 31–32 min 100–10%; 32–35 min 10% (all B in A, *v*/*v*). All UPLC (Acquit H-Class; Waters, Milford, MA, USA) analyses were performed using a YMC-Triart C_18_ column (100 × 2.1 mm, 1.9 μm, UV monitored at 280 nm) with a biphasic solvent system consisting of solvent B (ACN) and solvent A (H_2_O + 0.1% FA) flowing at a rate of 0.4 mL/min. The column temperature was set at 25°C, and the program was as follows: 0–0.5 min, 10%; 0.5–5.5 min, 10–100%; 5.5–5.6 min, 100%; 5.6–6 min, 100–10%; 6–6.5 min, 10% (all B in A, *v*/*v*). LC-HRMS was performed using a Bruker Impact HD Q-TOF mass spectrometer equipped with an electrospray ionization source. All analyses were performed under the same chromatographic conditions as those used for the HPLC analysis. The spectra were scanned in positive ion mode over the *m*/*z* range of 50–1000, with the source parameters set as follows: capillary voltage, 4500 V; end plate offset, -500 V; nebulizer pressure, 0.4 Bar; dry gas flow, 4.0 L/min; and dry temperature, 180°C. The HRMS data were processed using Bruker Compass Data Analysis version 4.2. The HRMS and UV spectra of all the compounds are presented in **Figs. S2 and S3**, respectively.

### 2.7 Bioconversion experiments

The *A. oryzae* transformants expressing tailoring enzymes were cultivated in 50 mL of CMP liquid medium at 30°C and 200 rpm for 1 day, then fed the corresponding precursors at a concentration of 50 mg/L, and subsequently incubated at 30°C and 200 rpm for an additional 3 days. Thereafter, the culture supernatant was extracted three times with ethyl acetate, and mycelia were extracted with methanol under ultrasonic conditions. Each extract of the organic phase was concentrated via vacuum distillation, redissolved, and further analyzed using HPLC according to the same procedures described in Section 2.6.

### 2.8 Fermentation, isolation, and purification of metabolites 1, 2, and 5–18

To acquire an adequate quantity of pure products, all *A. oryzae* transformants were subjected to large-scale fermentation in CMP medium, after which the filtered fermentation broth was extracted three times using an equal volume of ethyl acetate and further concentrated via vacuum distillation. Mycelia were extracted overnight with methanol at ambient temperature, concentrated, and re-extracted with ethyl acetate. Both extracts were then combined, separated on a Sephadex LH-20 column with methanol as the mobile phase, and further purification using semi-preparative HPLC (YMC-Pack Pro C18, 250 × 10.0 mm, 5 μm, UV monitored at 280 nm).

### 2.9 Expression and purification of recombinants Atn2 and Atn10

The fully constructed pET28b-*atn2* and pET28b-*atn10* plasmids were overexpressed in *E. coli* BL21 (DE3). A single colony was picked and cultured overnight at 37°C and 220 rpm in LB medium containing 50 μg/mL kanamycin. Subsequently, the seed culture was inoculated at a ratio of 1:100 into 1 L of LB medium containing 50 μg/mL kanamycin and grown at 37°C and 220 rpm for 3–6 h until the OD_600_ reached 0.6–0.8. Then, 0.2 mM IPTG was added to induce gene expression at 16°C and 150 rpm for 18–20 h. The cells were collected via centrifugation at 8000 *g* for 10 min, after which 10 g of cells resuspended in 40 mL of pre-cooled lysis buffer (50 mM NaH_2_PO_4_, 300 mM NaCl, 10 mM imidazole, and 10% glycerol, pH 8.0) was vortexed for complete mixing and then fragmented using a high-pressure homogenizer (NingBo Scientz Biotechnology Co., Ltd., China). The soluble fraction was collected via centrifugation at 10,000 *g* and 4°C for 1 h and incubated with 1–2 mL (depending on the protein yield) of Ni-NTA resins for 1–2 h at 4°C. The slurry was loaded onto an empty column, which was then washed with 300 mL of wash buffer (50 mM NaH_2_PO_4_, 300 mM NaCl, 20 mM imidazole, and 10% glycerol, pH 8.0) until no protein was detected in the flow-through. The protein fraction was then eluted with 1–5 mL of elution buffer (50 mM NaH_2_PO_4_, 300 mM NaCl, 250 mM imidazole, and 10% glycerol, pH 8.0) and concentrated using an Ultracel®-10/30K unit (Merck Millipore Ltd., Darmstadt, Germany) (determined by the target protein). After centrifugation at 4000 *g* and 4°C for 20 min, desalting buffer (50 mM NaH_2_PO_4_, 100 mM NaCl, and 10% glycerol, pH 7.4) was added for buffer exchange. Finally, 100 µL aliquots of the desalted fractions were flash-frozen with liquid nitrogen and store at -80°C until use. The purified Atn2 and Atn10 proteins were analyzed using SDS-PAGE, and the protein concentrations were determined using a NanoDrop™ One spectrophotometer (Thermo Fisher Scientific).

### 2.10 Enzymatic reaction assay of Atn2 and Atn10

All the *in vitro* assays described in this section were carried out in a total volume of 100 μL in the desalting buffer at 30°C for 2 h, and the boiled enzymes were used as negative controls unless otherwise specified. The enzymatic reaction of Atn2 with substrate **1** contained 2 µM of Atn2, 200 µM FeSO_4_, 5 mM α-ketoglutarate, 4 mM ascorbate, and 100 μM of the substrate, whereas the enzymatic reaction of Atn10 with substrate **1** comprised 2 μM Atn10, 5 mM NAD^+^, and 100 μM of the substrate. When Atn2 and Atn10 were reacted together with substrate **1**, the reaction system contained all the co-factors, 2 μM Atn2, 2 μM Atn10, and 100 μM of the substrate. The reactions were then quenched by thorough mixing with 300 μL of methanol to precipitate the proteins, and the mixtures were centrifuged at 12,000 *g* for 10 min to remove denatured proteins. Finally, the supernatants were analyzed using UPLC with the gradient elution program described in Section 2.6 applied.

### 2.11 Anti-inflammatory activity assay

Single-cell suspensions of RAW264.7 macrophages were prepared in culture medium containing 10% fetal bovine serum and then inoculated (∼1 × 10^6^ cells/mL in 90 μL) into the wells of 96-well plates and cultured at 37°C for 24 h under 5% CO_2_. Cell viability was evaluated using the CCK-8 assay. The samples to be tested were first dissolved in DMSO and diluted with basic culture medium and then 10 μL was added to each well of the 96-well plates. The following four groups were established: negative control (cells), LPS control (1 μg/mL LPS + cells), positive control (10 μM indomethacin + 1 μg/mL LPS), and drug (drug + LPS). After 24 h incubation, the cell supernatant in each well was transferred to a centrifuge tube and centrifuged for 3 min, after which the tube was sealed and stored at 4°C. Cell culture medium containing 10% CCK-8 was added to the wells of the culture plate from which the cell supernatants had been removed. After an appropriate time for color development, an enzyme-labeling instrument was used to measure the absorbance at 450 nm. The cell supernatant from the cells with greater than 80% viability (inhibition rate <20%) was used for NO determination (Griess method) [16,17], with the absorbance at 540 nm determined using the enzyme-labeling instrument. The formula for calculating NO inhibition was as follows: NO inhibition (%) = [(NO_LPS_ – NO_Drug_)/(NO_LPS_ – NO_Control_)] × 100%. The experimental results are expressed as the mean ± standard deviation.

### 2.12 Cytotoxicity assay

Eighteen human cancer cell lines (A549, MCF7, MKN-45, HepG2, HCT 116, SF126, HeLa, DU145, K-562, CAL-62, 786-O, PATU8988T, TE-1, HOS, 5637, A-375, GBC-SD, and A-673) and two human normal cell lines (L-02 and 293T) were provided by Wuhan Pricella Biotechnology Co., Ltd. (Wuhan, China). Cytotoxicity was evaluated using a previously established CCK-8 method, with cisplatin used as the positive control [18,19].

### 2.13 Antimicrobial activity assay

The antimicrobial activities of the isolated compounds against 12 pathogenic fungal strains (*Candida albicans* ATCC 10231, *Candida glabrata* ATCC 2001, *Sclerotinia sclerotiorum* (Lib.) de Bary, *Fusarium graminearum* Schw., *Phytophthora nicotianae, Bipolaris sorokiniana, Fusarium oxysporum* Schl., *Ceratobasidium graminearum, Alternaria panax, Valsa mali* Miyabe et Yamada, *Phytophthora infestans*, and *Lecanicillium lecanii*) and seven pathogenic bacterial strains (*Escherichia coli* ATCC 25922, *Staphylococcus aureus* ATCC 12600, *Bacillus subtilis* ATCC 6051, *Acinetobacter baumannii* ATCC 19606, *Enterococcus faecalis* ATCC 51559, *Klebsiella pneumoniae* subsp. *pneumoniae* ATCC 13883, and *Vibrio vulnificus* ATCC 27562) were evaluated at a final concentration of 50 μM using the broth microdilution method in 96-well sterile plates [20]. Ciprofloxacin (25 μM), ketoconazole (50 μM), and carbendazim (25 μM) were used as positive controls. The blank control consisted of a solvent and a blank culture medium, whereas the negative control comprised a solvent and a bacterial solution for co-cultivation, with DMSO serving as the solvent. The absorbance was measured at OD_600_ using a microplate reader. The results were compared with those of the blank and positive controls to assess the bacteriostatic activity of the compounds. The MIC was determined using a 12-point compound concentration gradient, which was sequentially prepared as follows: 50.00, 25.00, 12.50, 6.25, 3.13, 1.56, 0.78, 0.39, 0.20, 0.10, 0.05, and 0.02 μM. Three parallel wells were tested for each experiment and all experiments were repeated three times.

## 3 Results and discussion

### 3.1 Discovery and bioinformatic analysis of the atn gene cluster in Aspergillus versicolor ADS-F20

To identify the BGC of aspertetranones B (**1**) and D (**2**), we initially performed whole-genome sequencing analysis of the known producer, *A. versicolor* ADS-F20. In accordance with the established strategy for biosynthesizing fungal meroterpenoids, as exemplified by that for pyripyropene A [21,22], setosusin [23], and chrodrimanin B [24], the four fundamental enzymes that catalyze the formation of the intricate angular tetracyclic backbone structure must first be identified. Building on this, we initiated a comprehensive genomic BLAST search for the BGC using the probe gene PKS SetA. Subsequently, investigation of the flanking regions of the PKS gene allowed the discovery of the putative BGC of compound **1**, which was similar to that of ochraceopones (*och*) in *A. ochraceopetaliformis* SCSIO 05702 and was designated *atn* (Fig. 1b, **Table S4**). This cluster encodes four typical biosynthetic enzymes of meroterpenoids: the non-reducing polyketide synthase (NR-PKS) Atn1, the UbiA-like prenyltransferase (PT) Atn3, the FAD-dependent monooxygenase (FMO) Atn6, and the membrane-bound terpene cyclase (CYC) Atn9. These enzymes are expected to generate the first cyclized intermediate **6**. Furthermore, the *atn* cluster encodes eight putative enzymes, including four cytochrome P450s (Atn4, Atn5, Atn7, and Atn11), one phytanoyl-CoA dioxygenase (Atn2), a short-chain dehydrogenase/reductase (SDR) (Atn10), one nmrA-like reductase (Atn8), and one hypothetical isomerase (Atn12), which are responsible for the tailoring reactions and are involved in the biosynthesis of aspertetranones.

### 3.2 Enzymatic construction of angular tetracyclic derivatives by four core enzymes

**Fig. 2:**
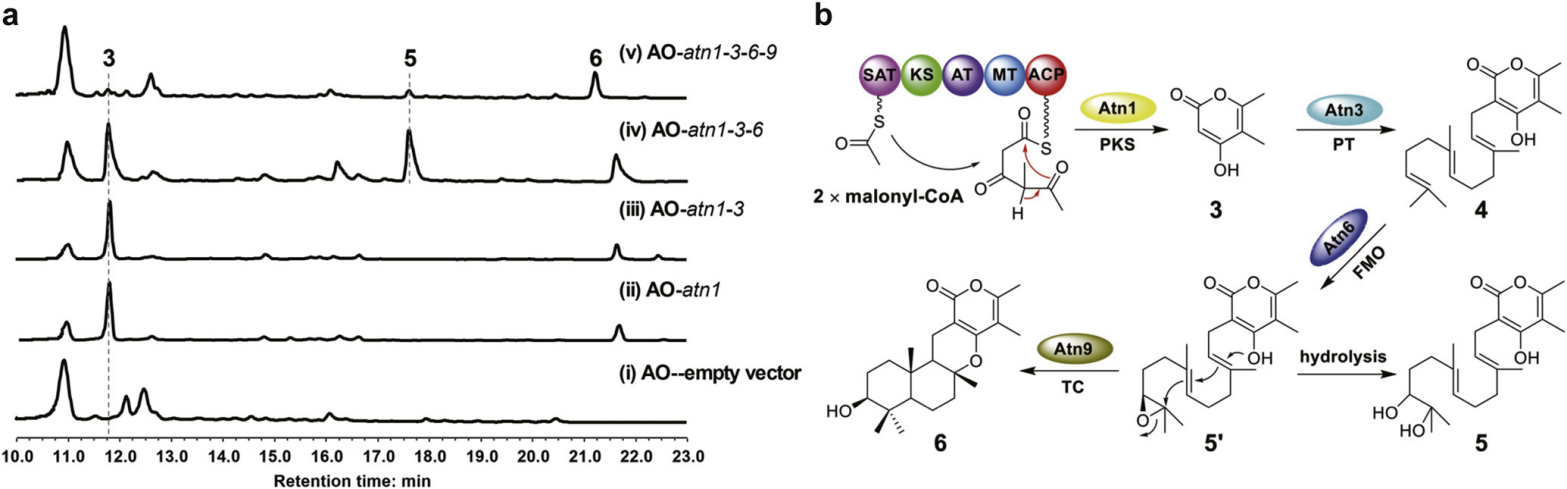
Heterologous reconstruction of compound **6** in *A. oryzae* NSAR1. (a) HPLC profiles of culture supernatant extracts from *A. oryzae* transformants harboring (i) the empty vector, (ii) *atn1*, (iii) *atn1*+*atn3*, (iv) *atn1*+*atn3*+*atn6*, and (v) *atn1*+*atn3*+*atn6*+*atn9*. The HPLC profiles were monitored at 280 nm. (b) Biosynthetic pathway of the angular tetracyclic intermediate **6**.

To accelerate the discovery and identification of novel derivatives related to the aspertetranone family, 12 genes in the *atn* cluster were heterologously expressed in *A. oryzae* NSAR1, a powerful platform for refactoring natural product biosynthesis in fungi [10]. Initially, the PKS gene *atn1* was solely expressed in *A. oryzae*, and the resulting transformant *Ao*-*atn1* was cultured in induction medium for 3 days. As expected, one new peak was detected compared with the HPLC profile of the wild-type strain without gene expression (Fig. 2a, **lanes i and ii**). The product was identified by HRMS (**Fig. S2**) and NMR analyses as 5-methyl TAL (**3**) (**Fig. S4**), confirming that Atn1 was a 5-methyl TAL synthase. Introduction of the prenyltransferase gene *atn3* into *A. oryzae* NSAR1 did not result in a new product in the culture supernatant extract (Fig. 2a, **lane iii**), but hydrophobic metabolite **4** was detected in the mycelial extract (**Fig. S5**), and its molecular formula was determined by HRMS analysis to be C_22_H_32_O_3_ (**4**, [M+H]^+^: *obsd.* 345.2436, *calcd.* 345.2430) (**Fig. S2**), indicating the incorporation of a farnesyl moiety.

Further introduction of the FMO gene *atn6* into the strain yielded the new product **5** (Fig. 2a, **lane iv**), a dihydroxy analog of compound **4** (**Fig. S6**) derived from the hydrolysis of the epoxide **5’**. On the basis of these results, we concluded that Atn3 acts as a 5-farnesyltransferase and that Atn6 is an FMO that forms an epoxide ring at the C-10 and C-11 positions of compound **4**. Finally, HPLC analysis revealed that the four-gene expression system, including the terpene cyclase gene *atn9*, had successfully generated the angular tetracyclic molecule **6** (Fig. 2a, **lane v**) with an *m/z* value of 361 [M+H]^+^ (**Fig. S2**). The structure was further determined using 1D and 2D NMR analyses (**Fig. S7**). These findings indicate that CYC Atn9 catalyzes the crucial cyclization of the prenyl group, a process initiated by protonation and the subsequent ring opening of the epoxide, thereby establishing a definitive biosynthetic pathway leading to the core structure of compound **1** (Fig. 2b).

### 3.3 Preliminary investigation into the substrate promiscuity of tailoring enzymes

**Fig. 3:**
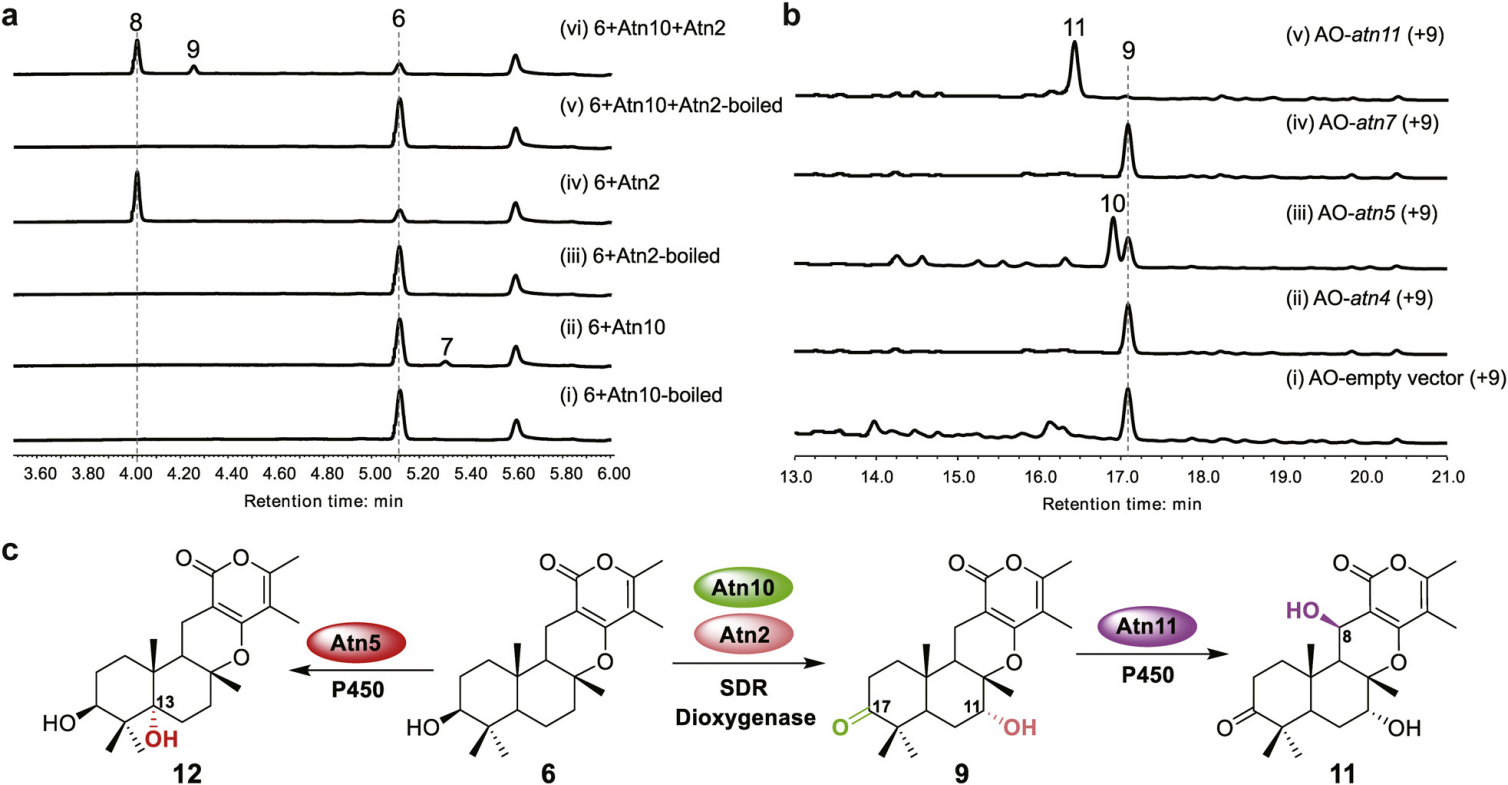
Catalytic order and functional characterization of tailoring enzymes. (a) UPLC profiles of the *in vitro* enzymatic reaction products of compound **6** catalyzed by Atn10 or Atn2, with boiled enzymes used as a negative control. (b) HPLC profiles of culture supernatant extracts from *A. oryzae* transformants incubated with compound **9**: transformants harboring (i) the empty vector, (ii) *atn4*, (iii) *atn5*, (iv) *atn7*, and (v) *atn11*. All the chromatograms were monitored at 280 nm. (c) Reactions catalyzed by Atn5 and Atn11.

Considering the expression characteristics and potential substrate promiscuity of the eight tailoring enzymes, we first screened their catalytic activities toward precursor **6**. Our initial investigations focused on Atn10 and Atn2. Given the abundance of closely related SDR homologs observed in fungal pathways of meroterpenoid biosynthesis, it is noteworthy that many of these SDRs can catalyze alcohol oxidation reactions at the C-17 position of products obtained from the cyclization process [25]. Additionally, Fe(II)/alpha-ketoglutarate (α-KG)-dependent dioxygenases play a crucial role in amplifying the structural diversity and complexity of the molecules [26]. Thus, *His*_6_-tagged Atn10 and Atn2 were recombinantly expressed in *E. coli* (DE3) and purified for *in vitro* biochemical assays (**Fig. S8**). HPLC analysis revealed that compound **6** was converted into products **7** and **8** by Atn10 and Atn2, respectively (Fig. 3a, **lanes i–iv**). Co-incubation of compound **6** with both Atn10 and Atn2 led to the disappearance of compound **7** and the formation of product **9**, a dehydrogenation product of compound **8** (Fig. 3a, **lanes v and vi**). In summary, Atn2 initiates the conversion of substrate **6** into compound **8**, which is further transformed into compound **9** by Atn10.

The membrane-bound nature of fungal P450s renders their heterologous expression in prokaryotic hosts such as *E. coli* difficult, but this is necessary for obtaining sufficient soluble and functional proteins for *in vitro* studies [27]. To investigate the catalytic order and functions of the four P450 enzymes, we performed feeding experiments in which compound **9** was added to the culture media of *A. oryzae* transformants expressing distinct tailoring enzymes. The host expressing *atn5* was capable of converting compound **9** into product **10** (Fig. 3b, **lanes i and iii**), which exhibited a 16 Da increase in molecular weight, suggesting that Atn5 catalyzes a hydroxylation reaction (Fig. 3c). Intriguingly, feeding compound **9** to the mutant *Ao*-*atn11* resulted in the generation of another product, compound **11** (Fig. 3b, **lane v**). LC-MS analysis confirmed that compound **11** was a hydroxylated product of compound **9** ([M+H]^+^: *obsd.* 391.2121, *calcd.* 391.2101) (**Fig. S2**), and its structure was identified using 1D and 2D NMR analyses (**Fig. S9**). However, compound **9** was not transformed by Atn4 or Atn7 (Fig. 3b, **lanes ii and iv**).

To further determine the substrate specificity of Atn5 and Atn11, compound **6** was fed to *Ao*-*atn5*, resulting in its efficient transformation into product **12** (**Fig. S10, lanes i and iii**) with a molecular weight of *m/z* 377 [M+H]^+^ (**Fig. S2**). Notably, the molecular weight of compound **12** was 16 Da greater than that of compound **6**, although the two exhibited similar UV-Vis spectra (**Fig. S3**). By contrast, the HPLC analysis showed no new peaks for the strain harboring *atn11* (**Fig. S10, lane iv**). Additionally, when precursor **6** was supplied to the six-gene strain *Ao*-*atn4-5-7-8-11-12*, a metabolic profile identical to that of *Ao*-*atn5* was observed (**Fig. S10, lanes ii and iii**). Interestingly, when precursor **9** was used, the metabolic profile matched that of *Ao*-*atn11* (**Fig. S10, lanes v and vi**). These findings clearly demonstrate that Atn5 functions before Atn11 in the main pathway. Accordingly, Atn2, Atn5, and Atn10 were assigned to the second module of the aspertetranone biosynthetic pathway.

### 3.4 Combination of Atn2, Atn5, and Atn10 for producing natural products

**Fig. 4:**
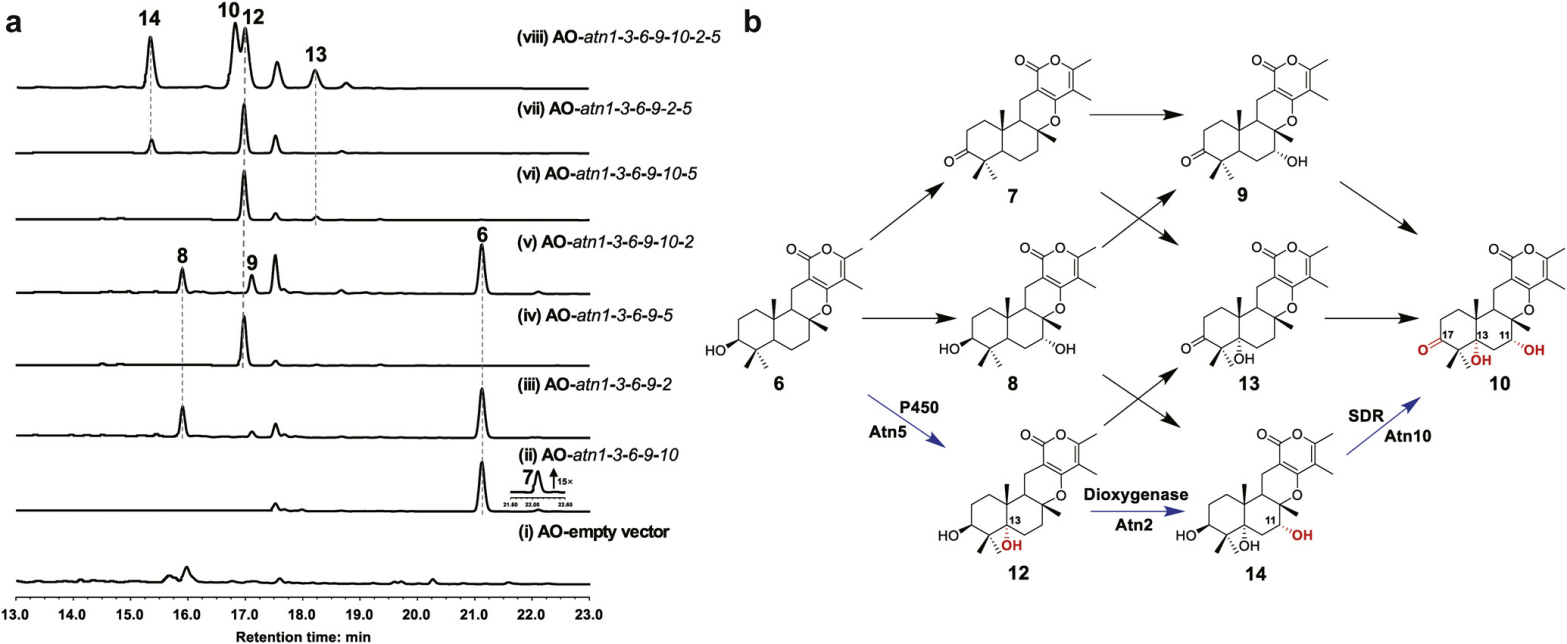
Metabolites derived from the catalytic network of the combinatorial expression of Atn2, Atn5, and Atn10. (a) HPLC profiles of culture supernatant extracts from *A. oryzae* transformants harboring (i) the empty vector, (ii) *atn1*+*atn3*+*atn6*+*atn9*+*atn10*, (iii) *atn1*+*atn3*+*atn6*+*atn9*+*atn2*, (iv) *atn1*+*atn3*+*atn6*+*atn9*+*atn5*, (v) *atn1*+*atn3*+*atn6*+*atn9*+*atn10*+*atn2*, (vi) *atn1*+*atn3*+*atn6*+*atn9*+*atn10*+*atn5*, (vii) *atn1*+*atn3*+*atn6*+*atn9*+*atn2*+*atn5*, and (viii) *atn1*+*atn3*+*atn6*+*atn9*+*atn10*+*atn2*+*atn5*. The chromatograms were monitored at 280 nm. (b) Six different identified pathways leading to the generation of compound **10** from substrate **6** catalyzed by Atn2, Atn5, and Atn10. The main pathway is shown in blue arrows.

Next, we mined and directionally synthesized a wide range of metabolites via cross-catalysis mediated by Atn10, Atn2, and Atn5. The three enzymes were individually introduced into four previously constructed gene-expressing transformants (*Ao*-*atn1-3-6-9*), and the resulting transformants generated the expected products **7, 8**, and **12**, respectively (Fig. 4a, **lanes ii–iv; and Figs. S11–S13**). According to the HPLC results, the efficiency of the three enzymes in catalyzing the reaction with substrate **6** was as follows: Atn5 exhibited the highest efficiency, followed by Atn2 and then Atn10. This suggests that compound **6** may act as a natural substrate of Atn5.

Additionally, we transferred these three genes in pairwise combinations into an *A. oryzae* strain capable of synthesizing **6**. The coexpression of Atn10 and Atn2 in strain *Ao*-*atn1-3-6-9-10-2* led to the appearance of three new peaks, labeled **6, 8**, and **9** (Fig. 4a, **lane v; and Fig. S14**), in the supernatant extract compared with the profile of control strain *Ao*-*atn1-3-6-9*. This observation was consistent with the results of our *in vitro* assays (Fig. 3a). Furthermore, the concurrent expression of Atn5 and Atn10 resulted in the formation of a new product, designated **11** (Fig. 4a, **lane vi**). The underlying biochemical process involves Atn5 hydroxylating compound **6** to form the intermediate **12**, which is further modified through oxidative dehydrogenation by Atn10 to yield compound **13** (**Fig. S15**). In another pairwise combination, the coexpression of Atn2 and Atn5 led to the synthesis of a new compound, designated as **14** (Fig. 4a, **lane vii; and Fig. S16**). In this pathway, Atn5 initially converts compound **6** into compound **12**, which is then converted into compound **14** by Atn2. Finally, Atn2, Atn5, and Atn10 were coexpressed in *Ao*-*atn1-3-6-9* to create the transformant *Ao*-*atn1-3-6-9-10-2-5*. HPLC analysis revealed the presence of an additional peak (identified as product **10**) (Fig. 4a, **lane viii; and Fig. S17**) compared with the HPLC profiles of the other strains. Furthermore, this strain produced varying quantities of the intermediates **12, 13**, and **14**.

Accordingly, the above-described results clearly demonstrate that under standardized *in vivo* bioconversion conditions, compound **10** can be synthesized via six distinct intersecting pathways (6→7→9→10; 6→7→13→10; 6→8→9→10; 6→8→14→10; 6→12→13→10; and 6→12→14→10) from the key intermediate **6** (Fig. 4b) because of the substrate promiscuity exhibited by Atn2, Atn5, and Atn10. Moreover, on the basis of the evaluation of enzymatic catalytic efficiency and the observed order of product generation, we speculate that **6→12→14→10** (Atn5→Atn2→Atn10) is the primary pathway in the natural biosynthetic process. Notably, the respective promiscuity of the three enzymes collectively contributed to the diversity of the precursors, and seven angular tetracyclic derivatives were produced through various structural modifications.

### 3.5 Diversification of products using additional tailoring enzymes

Subsequently, we reconstructed the complete biosynthetic process for compounds **1** and **2** with the aim of identifying as many intermediate derivatives as possible. Modification of the C-12 or C-15 sites is crucial for the conversion of compound **10** into the final product, and the remaining P450 enzymes catalyze this step. Consequently, we analyzed the bioconversion of compound **10** in single-gene transformants expressing *atn7* and *atn4*, respectively. The C12–C17 junction oxygen bridge product **15** was observed from the host expressing *atn7*, whereas Atn4 did not yield any new products (**Fig. S18, lanes i–iv**). The function of P450 Atn11 as a C-8 hydroxylase has been previously confirmed. Further feeding experiments showed that Atn11 could hydroxylate compound **10** to generate product **16** (**Fig. S18, lanes v and vi**), demonstrating its broad substrate promiscuity.

**Fig. 5:**
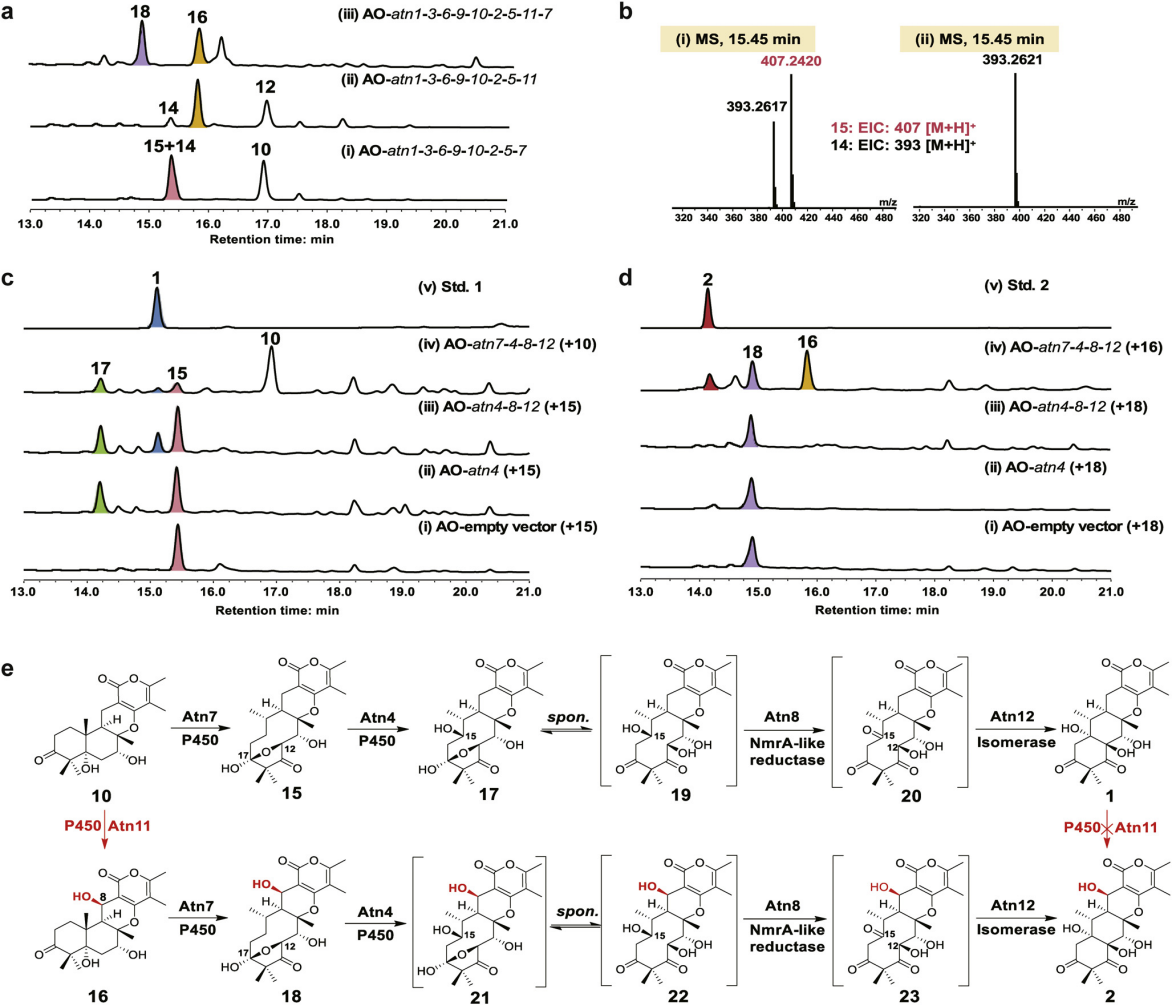
Heterologous biosynthesis of end-products **1** and **2**. (a) HPLC profiles of culture supernatant extracts from *A. oryzae* transformants harboring (i) *atn1*+*atn3*+*atn6*+*atn9*+*atn10* +*atn2*+*atn5*+*atn7*, (ii) *atn1*+*atn3*+*atn6*+*atn9*+*atn10*+*atn2*+*atn5*+*atn11*, and (iii) *atn1*+*atn3*+*atn6*+*atn9*+*atn10*+*atn2*+*atn5*+*atn11*+*atn7*. (b) LC-HRMS profiles recorded over 15.45 min for (i) *Ao*-*atn1-3-6-9-10-2-5-7* and (ii) *Ao*-*atn1-3-6-9-10-2-5-11*. EICs of compounds **15** (*m/z* = 407 [M+H]^+^) and **14** (*m/z* = 393 [M+H]^+^). (c) Bioconversion analysis using *A. oryzae* transformants expressing selected enzymes. Profiles from feeding compound **15** to transformants expressing (i) the empty vector, (ii) *atn4*, and (iii) *atn4*+*atn8*+*atn12*; (iv) profile from feeding compound **10** to the transformant expressing *atn7*+*atn4*+*atn8*+*atn12*; and (v) profile of the standard of compound **1**. (d) Profiles from feeding compound **18** to transformants expressing (i) the empty vector, (ii) *atn4*, and (iii) *atn4*+*atn8*+*atn12*; (iv) profile from feeding compound **16** to the transformant expressing *atn7*+*atn4*+*atn8*+*atn12*; and (v) profile of the standard of compound **2**. All chromatograms were monitored at 280 nm. (e) Proposed biosynthetic pathways for substrates **1** and **2**.

Additionally, two multi-gene transformants were successfully constructed by incorporating Atn7 and Atn11 into the strain producing compound **10**, respectively. HPLC analysis revealed that both strains produced the expected products **15** and **16** (Fig. 5a, **lanes i and ii**), which were subsequently validated via 1D and 2D NMR analyses (**Figs. S19 and S20**). Notably, the retention times of products **15** and **14** coincided (*t_R_* = 15.45 min) in the chromatogram, suggesting their potential polar similarity. Moreover, LC-HRMS analysis revealed distinctive differences in the ion fragment profiles. Specifically, an ion peak at *m/z* 393 for product **14** was observed in the extracts from the *Ao-atn1-3-6-9-10-2-5-11* culture, whereas dual ion peaks at *m/z* 407 and 393, corresponding to products **15** and **14**, respectively (Fig. 5b), were detected in strain *Ao*-*atn1-3-6-9-10-2-5-7*. Furthermore, Atn11 failed to convert product **15**, and compound **18** was generated by introducing *atn7* into strain *Ao-atn1-3-6-9-10-2-5-11* (Fig. 5a, **lane iii; and Fig. S21**), suggesting that Atn11 must function before Atn7 does in the main pathway.

Having encountered difficulties in establishing the 12-gene expression system, likely due to the expression abandons of multiple genes, we instead performed a bioconversion analysis using the combinatorial expression of Atn4, Atn8, and Atn12 in *A. oryzae*. The hosts coexpressing *atn4, atn8*, and *atn12* successfully converted compound **15** into two products. One of these peaks, product **17**, had a molecular mass that was 16 Da higher than that of product **12** ([M+H]^+^: *obsd.* 423.2014, *calcd.* 423.2019) (**Fig. S2**), suggesting that the hydroxylation reaction was catalyzed by one of the three enzymes. Subsequent feeding experiments with single-gene strains confirmed that Atn4 was responsible for the production of compound **17**. The other product was identified as compound **1** (Fig. 5c, **lanes iii and v**) with an expected *m/z* value of 421 [M+H]^+^ (**Fig. S2**). Additionally, the product of Atn8 was not detected, suggesting that it is an unstable intermediate.

Since Atn11 failed to catalyze the transformation of compound **1** into compound **2** (**Fig. S22**), substrate **18** was selected as the entry point for the directed biosynthesis of compound **2**. However, the transformants *Ao*-*atn4* and *Ao*-*atn4-8-12* failed to achieve this conversion (Fig. 5d, **lanes i–iii**). Interestingly, this trend differed from that of precursor **15**, suggesting that modifications at the C-8 position of compound **18** alter its intermolecular interactions with Atn4, thereby affecting the catalytic activity. Finally, a four-gene-expressing host harboring *atn7, atn4, atn8*, and *atn12* successfully converted compound **16** into the end-product **2** (**Fig. S23**), and intermediate **18** was observed in the extracts (Fig. 5d, **lane iv**). Feeding experiments with substrate **10** yielded the expected metabolites **15, 17**, and **1** (Fig. 5c, **lane iv**). Although the biosyntheses of compounds **2** and **1** were mediated by the same four enzymes, substrate recognition differed between their respective pathways (Fig. 5e). Despite that authentic intermediates were not obtained, our results indicated a tight connection among tailoring enzymes within the gene cluster, suggesting a dynamic and variable substrate conversion process.

### 3.6 Evaluation of biological activities

Finally, we evaluated the pharmacological profiles of the 16 isolated meroterpenoids to guide future therapeutic development. Aspertetranones have been reported to show anti-inflammatory activity. Thus, the NO inhibitory activities of all metabolites were tested using LPS-stimulated RAW264.7 macrophages. However, none of the tested compounds exhibited significant NO inhibitory activity at 50 μM, showing markedly lower efficacy than that of the positive control, indomethacin (**Table S6**).

**Fig. 6:**
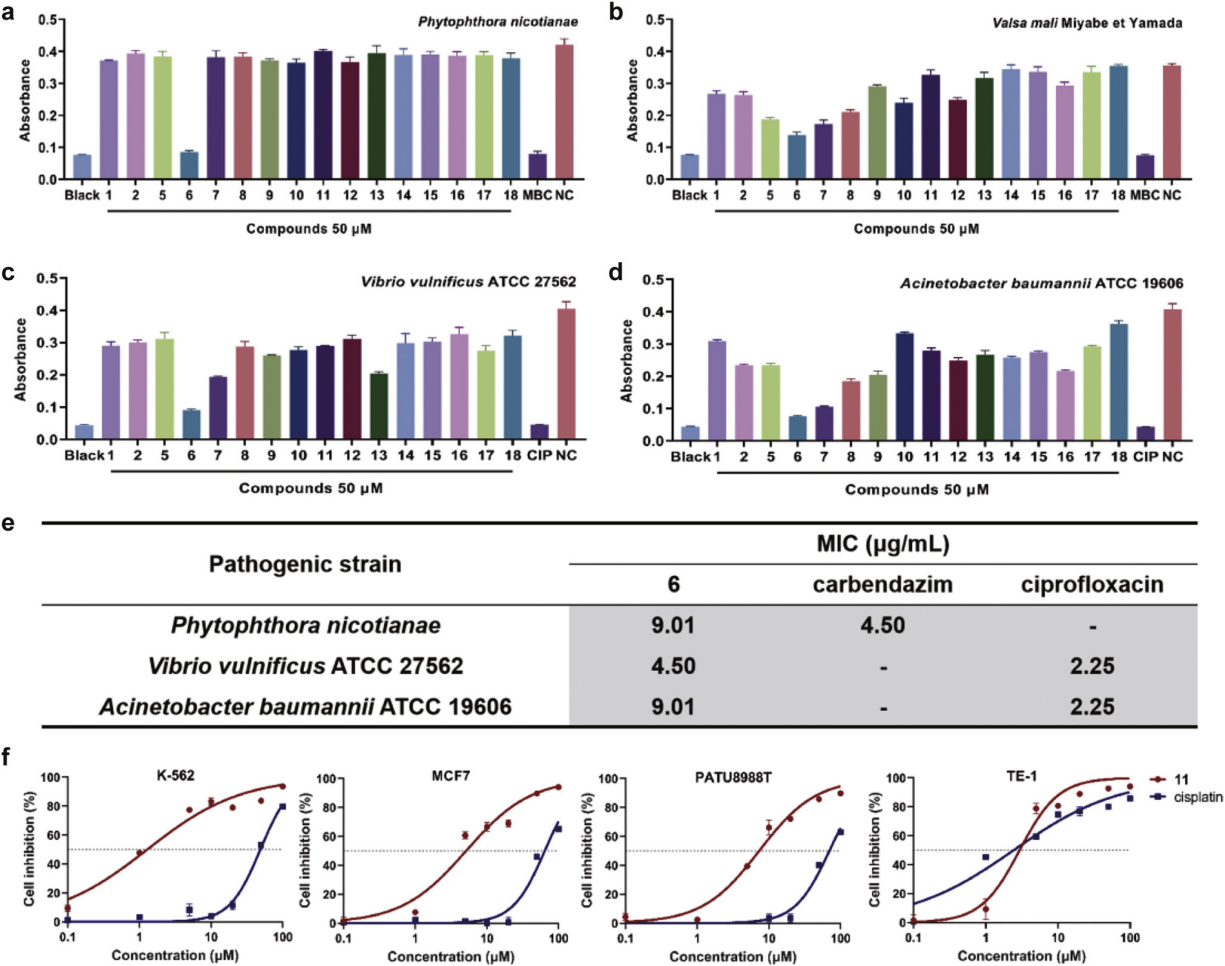
Antimicrobial activities of tested compounds against (a) *Phytophthora nicotianae*, (b) *Valsa mali* Miyabe et Yamada, (c) *Vibrio vulnificus* ATCC 27562, and (d) *Acinetobacter baumannii* ATCC 19606. (e) MIC values of compound **6**. (f) Dose–response curves of the antiproliferative activities of compound **11** and cisplatin toward K-562, MCF7, PATU8988T, and TE-1 cells. All inhibition and cytotoxicity data in this figure are the mean ± SD; *n* = 3 biologically independent replicates. MBC: carbendazim; CIP: ciprofloxacin; NC: negative control. MIC values for compound **6** are shown in **Table S9**. The dose–response curves used to generate IC_50_ values for these compounds are shown in **Table S11**.

Additionally, all compounds were tested for antimicrobial activity toward 12 pathogenic fungi and seven pathogenic bacteria. The results revealed that compound **6** exhibited significant antifungal activity toward *Phytophthora nicotianae*, with an MIC value of 9.01 μg/mL (Fig. 6a). Compounds **5**–**8** showed inhibitory effects against *Valsa mali* Miyabe et Yamada, and all tested compounds were effective against *Sclerotinia sclerotiorum* (Lib.) de Bary, albeit they did not completely inhibit the growth of these fungi (Fig. 6b). Intriguingly, the inhibitory activity of the tetracyclic derivatives against *V. mali* demonstrated a marked structural dependence, with the order of efficacy being **6** > **7** > **8** > **10** > **9** > **11**. This indicates that the C-17 hydroxyl group in compound **6** confers the most significant positive contribution to its antifungal activity. Conversely, the sequential introduction of a ketone moiety at C-17 and hydroxyl groups at C-11, C-13, and C-8 results in a consistent decrease in activity. Notably, the growth of *Vibrio vulnificus* ATCC 27562 and *Acinetobacter baumannii* ATCC 19606 was effectively inhibited by compound **6**, with MIC values of 4.50 and 9.01 μg/mL, respectively (Fig. 6c–e). Moreover, compounds **7** and **8** also exhibited antibacterial activity against *A. baumannii* and *V. vulnificus*. Our results also indicated that the remaining compounds showed weak or inactive antimicrobial activity toward the other pathogenic strains at 50 μM concentration (**Tables S7–S9**).

The cytotoxic potential of all isolated compounds was screened across a panel of 20 diverse cancer cell lines (A549, MCF7, MKN-45, HepG2, HCT 116, SF126, HeLa, DU145, K-562, CAL-62, 786-O, PATU8988T, TE-1, HOS, 5637, A-375, GBC-SD, A-673, L-02, and 293T), with IC_50_ values determined for compounds exhibiting greater than 50% cell growth inhibition (**Table S10**). Despite its significant antimicrobial activity, compound **6** exhibited limited toxicity toward 5637 and 293T cells, with IC_50_ values of 41.55 and 44.92 μM, respectively. Most strikingly, compound **11** exhibited potent and selective toxicity against K-562, MCF7, and PATU8988T cells, with IC_50_ values (1.29, 5.12, and 7.34 μM, respectively) that were substantially lower than those of the positive control cisplatin (48.68, 64.19, and 69.80 μM, respectively). Furthermore, compound **11** also displayed significant toxicity toward A549, MKN-45, HCT 116, TE-1, and L-02 cells (IC_50_ = 4.10, 3.43, 3.13, 2.95, and 2.15 μM, respectively), with efficacy approaching that of cisplatin (Fig. 6f, **Table S11**). The other tested compounds showed no cytotoxic effects at 50 μM concentration. These findings indicate that the C-8 hydroxyl group, introduced via the P450 Atn11-catalyzed oxidation of compound **11**, is crucial for the toxicity of the compound toward cancer cell lines. Taken together, our screening results suggest that the angular tetracyclic compounds within this series of meroterpenoids exhibit superior biological activities compared with their linear tetracyclic analogs.

## 4 Conclusions and future prospects

In this study, we successfully identified and functionally characterized the *atn* BGC responsible for producing aspertetranones in deep-sea *A. versicolor* ADS-F20. Through heterologous expression of the *atn* genes in *A. oryzae* NSAR1, we not only reconstructed the native pathway but, more importantly, also demonstrated the power of pathway reprogramming to achieve the targeted biosynthesis of 17 derivatives. This chemical diversification is a direct consequence of the intrinsic substrate promiscuity of the key tailoring enzymes within the network. Specifically, enzymes such as the P450s (Atn5, Atn7, and Atn11), Fe(II)/α-KG-dependent dioxygenase (Atn2), and SDR (Atn10) act as flexible nodes, being capable of modifying multiple related intermediates. By strategically combining these enzymes, we effectively engineered the catalytic network and redirected the metabolic flux to generate both expected and novel shunt products, thereby elucidating the precise enzymatic logic governing their complex assembly.

The evolutionary plasticity of the aspertetranone scaffold, as evidenced by its tolerance to various enzymatic modifications, provides a mechanistic foundation for our success. This plasticity suggests that nature may utilize similar combinatorial strategies to generate chemical diversity in marine fungi. The broad-spectrum antimicrobial and anticancer activities revealed in our biological evaluation further validated this approach, revealing clear structure–activity relationships and highlighting the untapped pharmacological potential of this chemical family.

Furthermore, the engineered catalytic network established in this study not only provides a route to biosynthesizing aspertetranones but also establishes a versatile synthetic biology platform. The “plug-and-play” nature of our system, wherein individual enzymes or modules can be swapped or introduced, can be readily adapted for the diversification of other fungal meroterpenoid scaffolds. The ability to rapidly generate a focused library of structurally complex bioactive derivatives makes this platform a powerful tool for future drug discovery. In conclusion, by integrating pathway reprogramming and catalytic network engineering, our work not only expands the accessible chemical space of fungal meroterpenoids but also provides a robust and generalizable strategy for unlocking the therapeutic potential hidden within microbial genomes.

## Data availability statement

The data that support the findings of this study are available in the supplementary material of this article.

## CRediT authorship contribution statement

**Peiyuan Feng:** Writing – original draft, Project administration, Methodology, Investigation. **Moli Sang:** Investigation, Data curation, Conceptualization. **Wei Zhang:** Writing – review & editing, Validation, Supervision, Project administration, Methodology, Investigation, Funding acquisition, Formal analysis, Data curation, Conceptualization.

## Declaration of competing interest

The authors declare that they have no known competing financial interests or personal relationships that could have appeared to influence the work reported in this paper.

## Appendix Supplementary materials

Image, application 1

Image, application 2
