# Supplementary material for: Pathway reprogramming and catalytic network engineering for the production of bioactive aspertetranones from deep-sea Aspergillus versicolor ADS-F20
Source: Eng Microbiol. 2026 Jan 2;6(2):100259. doi: 10.1016/j.engmic.2025.100259 (PMC13323904; doi:10.1016/j.engmic.2025.100259)
Supplement: Supplementary file 1 [file mmc1.docx]

**Supplementary Information**

**Pathway reprogramming and catalytic network engineering for the production of bioactive aspertetranones from deep-sea *Aspergillus versicolor* ADS-F20**

Peiyuan Feng^[a] [b]^, Moli Sang^[a] [b]^, Wei Zhang^[a] [b] [c] [d]*^

^[a]^ Laboratory of Experimental Marine Biology, Institute of Oceanology, Chinese Academy of Sciences, Qingdao 266000, China

^[b]^ State Key Laboratory of Microbial Technology, Shandong University, Qingdao, Shandong, 266237, China

^[c]^ Laboratory for Marine Biology and Biotechnology, Qingdao Marine Science and Technology Center, Qingdao, Shandong, 266237, China

^[d]^ Shenzhen Research Institute of Shandong University, Shenzhen, 518057, China

* To whom correspondence should be addressed: weizhang@qdio.ac.cn (W.Z.)

**Contents**

**[Supplementary Tables](#_Toc28616)** [4](#_Toc28616)

**[Table S1.](#_Toc582)** [Primers used in this study. 4](#_Toc582)

**[Table S2.](#_Toc20927)** [Plasmids constructed in this study and PCR/ligation conditions. 5](#_Toc20927)

**[Table S3.](#_Toc32551)** [Transformants of](#_Toc32551) *[Aspergillus oryzae](#_Toc32551)* [NSAR1 constructed in this study. 6](#_Toc32551)

**[Table S4.](#_Toc20839)** [Annotation of each protein in the](#_Toc20839) *[atn](#_Toc20839)* [cluster. 7](#_Toc20839)

**[Table S5.](#_Toc366)** [NMR data and structure. 8](#_Toc366)

**[Supplementary Figures](#_Toc6767)** [15](#_Toc6767)

**[Fig. S1.](#_Toc15322)** [Analysis of the](#_Toc15322) *[Aspergillus versicolor](#_Toc15322)* [ADS-F20 fermentation products. 15](#_Toc15322)

**[Fig. S2.](#_Toc6977)** [The HRESIMS spectra of the compounds in the positive ion mode in this study. 16](#_Toc6977)

**[Fig. S3.](#_Toc22341)** [The UV-Vis spectra of the compounds in this study. 17](#_Toc22341)

**[Fig. S4.](#_Toc25853)** [NMR spectra of compound](#_Toc25853) **[3](#_Toc25853)** [(DMSO-](#_Toc25853)*[d](#_Toc25853)_[6](#_Toc25853)_*[). 18](#_Toc25853)

**[Fig. S5.](#_Toc17721)** [HPLC profiles of mycelial extracts from](#_Toc17721) *[A. oryzae](#_Toc17721)* [transformants 19](#_Toc17721)

**[Fig. S6.](#_Toc23625)** [NMR spectra of compound](#_Toc23625) **[5](#_Toc23625)** [(DMSO-](#_Toc23625)*[d](#_Toc23625)_[6](#_Toc23625)_*[). 20](#_Toc23625)

**[Fig. S7.](#_Toc13310)** [NMR spectra of compound](#_Toc13310) **[6](#_Toc13310)** [(CD](#_Toc13310)_[3](#_Toc13310)_[OD). 21](#_Toc13310)

**[Fig. S8.](#_Toc18845)** [The SDS-PAGE analysis of purified N-His](#_Toc18845)_[6](#_Toc18845)_[-tagged recombinant proteins Atn10 (27 kDa) and Atn2 (32.69 kDa). 22](#_Toc18845)

**[Fig. S9.](#_Toc6199)** [NMR spectra of compound](#_Toc6199) **[11](#_Toc6199)** [(DMSO-](#_Toc6199)*[d](#_Toc6199)_[6](#_Toc6199)_*[). 23](#_Toc6199)

**[Fig. S10.](#_Toc3317)** [The bioconversion analysis of](#_Toc3317) **[6](#_Toc3317)** [and](#_Toc3317) **[9](#_Toc3317)** [with](#_Toc3317) *[A. oryzae](#_Toc3317)* [transformants expressing one of the](#_Toc3317) *[atn](#_Toc3317)* [genes. 24](#_Toc3317)

**[Fig. S11.](#_Toc8266)** [NMR spectra of compound](#_Toc8266) **[7](#_Toc8266)** [(DMSO-](#_Toc8266)*[d](#_Toc8266)_[6](#_Toc8266)_*[). 25](#_Toc8266)

**[Fig. S12.](#_Toc6159)** [NMR spectra of compound](#_Toc6159) **[8](#_Toc6159)** [(DMSO-](#_Toc6159)*[d](#_Toc6159)_[6](#_Toc6159)_*[). 26](#_Toc6159)

**[Fig. S13.](#_Toc18470)** [NMR spectra of compound](#_Toc18470) **[12](#_Toc18470)** [(DMSO-](#_Toc18470)*[d](#_Toc18470)_[6](#_Toc18470)_*[). 27](#_Toc18470)

**[Fig. S14.](#_Toc11167)** [NMR spectra of compound](#_Toc11167) **[9](#_Toc11167)** [(DMSO-](#_Toc11167)*[d](#_Toc11167)_[6](#_Toc11167)_*[). 28](#_Toc11167)

**[Fig. S15.](#_Toc14377)** [NMR spectra of compound](#_Toc14377) **[13](#_Toc14377)** [(CD](#_Toc14377)_[3](#_Toc14377)_[OD). 29](#_Toc14377)

**[Fig. S16.](#_Toc30496)** [NMR spectra of compound](#_Toc30496) **[14](#_Toc30496)** [(CD](#_Toc30496)_[3](#_Toc30496)_[OD). 30](#_Toc30496)

**[Fig. S17.](#_Toc12330)** [NMR spectra of compound](#_Toc12330) **[10](#_Toc12330)** [(DMSO-](#_Toc12330)*[d](#_Toc12330)_[6](#_Toc12330)_*[). 31](#_Toc12330)

**[Fig. S18.](#_Toc30762)** [The bioconversion analysis of](#_Toc30762) **[10](#_Toc30762)** [with](#_Toc30762) *[A. oryzae](#_Toc30762)* [transformants expressing P450s. 32](#_Toc30762)

**[Fig. S19.](#_Toc2047)** [NMR spectra of compound](#_Toc2047) **[15](#_Toc2047)** [(DMSO-](#_Toc2047)*[d](#_Toc2047)_[6](#_Toc2047)_*[). 33](#_Toc2047)

**[Fig. S20.](#_Toc7539)** [NMR spectra of compound](#_Toc7539) **[16](#_Toc7539)** [(CD3OD). 34](#_Toc7539)

**[Fig. S21.](#_Toc22766)** [NMR spectra of compound](#_Toc22766) **[18](#_Toc22766)** [(DMSO-](#_Toc22766)*[d](#_Toc22766)_[6](#_Toc22766)_*[). 35](#_Toc22766)

**[Fig. S22.](#_Toc4840)***[In vivo](#_Toc4840)* [bioconversion analysis between end products](#_Toc4840) **[1](#_Toc4840)** [and](#_Toc4840) **[2](#_Toc4840)**[. 36](#_Toc4840)

**[Fig. S23.](#_Toc16362)** [NMR spectra of compound](#_Toc16362) **[2](#_Toc16362)** [(DMSO-](#_Toc16362)*[d](#_Toc16362)_[6](#_Toc16362)_*[). 37](#_Toc16362)

Supplementary Tables

**Table S1.** Primers used in this study.

| Primer | Sequence (5' to 3') |
| --- | --- |
| Atn1-F | CGAATTCGAGCTCGGTAatgtctccgaactggccctg |
| Atn1-R | CTACTACAGATCCCCGGGctacgctcgtctagccacaa |
| Atn2-F | gcaagctccgaattcatgacagccgatcctaccc |
| Atn2-R | ggccgcgctagcgaattcctacacttcaagtggttc |
| Atn3-F | cgcggcagcgagctccatatgatggctctcctccggga |
| Atn3-R | ttaagagatctctcgagcatatgctacccagaaaacacc |
| Atn4-F | gcggcagcgagctccatatgatgaagcttcacctcat |
| Atn4-R | agagatctctcgagcatatgtgatgcaagtagattct |
| Atn5-F | cagcaagctccgaattcatgccagaccagatatc |
| Atn5-R | ccgcgctagcgaattcctatcccggtcccagtgtta |
| Atn6-F | gcggcagcgagctccatatgatggcaaactccggtt |
| Atn6-R | taagagatctctcgagcatatgctacctcgaaataaca |
| Atn7-F | gcaagctccgaattcatggggtcatctcatattga |
| Atn7-R | cgcgctagcgaattcctatgtcatcctctcacgga |
| Atn8-F | gcggcagcgagctccatatgatgaataatgacatcacc |
| Atn8-R | cccttaagagatctctcgagctagttcttctcaagtgc |
| Atn9-F | cacagcaagctccgaattcatgaaccctaccggtac |
| Atn9-R | gcggccgcgctagcgaattcctacttcgctttcgac |
| Atn10-F | cggcagcgagctccatatgatgtcaacagtgcgat |
| Atn10-R | agatctctcgagcatatgctaaacagtggaccaggca |
| Atn11-F | ggcagcgagctccatatgatgcaaagctacggtatg |
| Atn11-R | gatctctcgagcatatgttatgtcttcctccgccgca |
| Atn12-F | gcgcggcagcgagctccatatgatgggctccatctcaac |
| Atn12-R | cccttaagagatctctcgagctaacagtccttggtcct |
| Atn6-F2 | cggccagtgccaagcttgcatgtcatggtgttttgatcat |
| Atn6-R2 | agactctagagtcgacctgcaggctggaaagcgggcagtg |
| Atn9-F2 | gctttccagcctgcaggtcatggtgttttgatcat |
| Atn9-R2 | gcagactctagagtcgactggaaagcgggcagtg |
| Atn7-F2 | cactgcccgctttccagcctgcaggtcatggtgttttgat |
| Atn7-R2 | atttaaaatgatcaaaacaccatgactggaaagcgggcag |
| pAdeA-F | CTATAGGGAAAGCTTGCATGttaattccgttcctttgc |
| pAdeA-R | CTCTAGAGTCGACCTGCAGGtcatggtgttttgatcat |
| pAdeA-F2 | aaaacaccatgaCCTGCAGGctggaaagcgggcag |
| pAdeA-R2 | AGTAGATCCTCTAGAGTCGAtcatggtgttttgatcat |
| pAdeA-F3 | tgaTCGACTCTAGAGGATCTctggaaagcgggcagt |
| pAdeA-R3 | TGTCCCCAATCCATATGACTAGTtcatggtgttttgatc |
| pAdeA-F4 | taaaatgatcaaaacaccatgactggaaagcgggcagtg |

**Table S2.** Plasmids constructed in this study and PCR/ligation conditions.

| Plasmid | Insert | Vector | Primer 1 | Primer 2 | PCR template |
| --- | --- | --- | --- | --- | --- |
| pUSA-*atn1* | *atn1* | pUSA digested with *Sma* I | Atn1-F | Atn1-R | gDNA |
| pTAex3-*atn2* | *atn2* | pTAex3 digested with *Eco*R I | Atn2-F | Atn2-R | gDNA |
| pTAex3-*atn3* | *atn3* | pTAex3 digested with *Nde* I | Atn3-F | Atn3-R | gDNA |
| pTAex3-*atn4* | *atn4* | pTAex3 digested with *Nde* I | Atn4-F | Atn4-R | gDNA |
| pTAex3-*atn5* | *atn5* | pTAex3 digested with *Eco*R I | Atn5-F | Atn5-R | gDNA |
| pTAex3-*atn6* | *atn6* | pTAex3 digested with *Nde* I | Atn6-F | Atn6-R | gDNA |
| pTAex3-*atn7* | *atn7* | pTAex3 digested with *Eco*R I | Atn7-F | Atn7-R | gDNA |
| pTAex3-*atn8* | *atn8* | pTAex3 digested with *Nde* I | Atn8-F | Atn8-R | gDNA |
| pTAex3-*atn9* | *atn9* | pTAex3 digested with *Eco*R I | Atn9-F | Atn9-R | gDNA |
| pTAex3-*atn10* | *atn10* | pTAex3 digested with *Nde* I | Atn10-F | Atn10-R | gDNA |
| pTAex3-*atn11* | *atn11* | pTAex3 digested with *Nde* I | Atn11-F | Atn11-R | gDNA |
| pTAex3-*atn12* | *atn12* | pTAex3 digested with *Nde* I | Atn12-F | Atn12-R | gDNA |
| pTAex3-*atn3*+*6* | *PamyB-atn6-TamyB* | pTAex3-*atn3* digested with *Sbf* I | Atn6-F2 | Atn6-R2 | pTAex3-*atn6* |
| pTAex3-*atn3*+*6*+*9* | *PamyB-atn9-TamyB* | pTAex3-*atn3+6* digested with *Sbf* I | Atn9-F2 | Atn9-R2 | pTAex3-*atn9* |
| pTAex3-atn*4*+*8* | *PamyB-atn8-TamyB* | pTAex3-*atn4* digested with *Sbf* I | Atn6-F2 | Atn6-R2 | pTAex3-*atn8* |
| pTAex3-atn*4*+*8*+*12* | *PamyB-atn12-TamyB* | pTAex3-*atn4*+*8* digested with *Sbf* I | Atn9-F2 | Atn9-R2 | pTAex3-*atn12* |
| pTAex3-atn*7*+*4*+*8*+*12* | *PamyB-atn7-TamyB* | pTAex3-*atn4*+*8*+*12* digested with *Sbf* I | Atn7-F2 | Atn7-R2 | pTAex3-*atn7* |
| pAdeA-*atn2* | *PamyB-atn2-TamyB* | pAdeA digested with *Sbf* I | pAdeA-F | pAdeA-R | pTAex3-*atn2* |
| pAdeA-*atn5* | *PamyB-atn5-TamyB* | pAdeA digested with *Sbf* I | pAdeA-F | pAdeA-R | pTAex3-*atn5* |
| pAdeA-*atn10* | *PamyB-atn10-TamyB* | pAdeA digested with *Sbf* I | pAdeA-F | pAdeA-R | pTAex3-*atn10* |
| pAdeA-*atn2+10* | *PamyB-atn2-TamyB* | pAdeA-*atn10* digested with *Sbf* I | pAdeA-F2 | pAdeA-R2 | pTAex3-*atn2* |
| pAdeA-*atn2+5* | *PamyB-atn2-TamyB* | pAdeA-*atn5* digested with *Sbf* I | pAdeA-F2 | pAdeA-R2 | pTAex3-*atn2* |
| pAdeA-*atn5+10* | *PamyB-atn10-TamyB* | pAdeA-*atn5* digested with *Sbf* I | pAdeA-F2 | pAdeA-R2 | pTAex3-*atn10* |
| pAdeA-*atn2+10+5* | *PamyB-atn5-TamyB* | pAdeA-*atn2+10* digested with *Spe* I | pAdeA-F3 | pAdeA-R3 | pTAex3-*atn5* |
| pAdeA-*atn2+10+5+7* | *PamyB-atn7-TamyB* | pAdeA-*atn2+10*+5 digested with *Spe* I | pAdeA-F4 | pAdeA-R3 | pTAex3-*atn7* |
| pAdeA-*atn2+10+5+11* | *PamyB-atn11-TamyB* | pAdeA-*atn2+10*+5 digested with *Spe* I | pAdeA-F4 | pAdeA-R3 | pTAex3-*atn11* |
| pAdeA-*atn2+10+5+11*+*7* | *PamyB-atn11-TamyB* | pAdeA-*atn2+10*+5+7 digested with *Spe* I | Atn6-F2 | pAdeA-F4 | pTAex3-*atn11* |
| pET28b-*atn2* | *atn2* | pET28b digested with *Nde* I and *Xho* I |  |  |  |
| pET28b-*atn10* | *atn10* | pET28b digested with *Nde* I and *Xho* I |  |  |  |

**Table S3.** Transformants of *Aspergillus oryzae* NSAR1 constructed in this study.

| Strain | Host strain | Plasmids used for transformation |
| --- | --- | --- |
| *AO-atn1* | *A. oryzae* NSAR1 | pUSA-*atn1* |
| *AO-atn4* | *A. oryzae* NSAR1 | pTAex3-*atn4* |
| *AO-atn5* | *A. oryzae* NSAR1 | pTAex3-*atn5* |
| *AO-atn7* | *A. oryzae* NSAR1 | pTAex3-*atn7* |
| *AO-atn11* | *A. oryzae* NSAR1 | pTAex3-*atn11* |
| *AO-atn1*-*3* | *A. oryzae* NSAR1 | pUSA-*atn1*, pTAex3-*atn3* |
| *AO-atn1*-*3*-*6* | *A. oryzae* NSAR1 | pUSA-*atn1*, pTAex3-*atn3*+*6* |
| *AO-atn1*-*3*-*6*-*9* | *A. oryzae* NSAR1 | pUSA-*atn1*, pTAex3-*atn3*+*6*+*9* |
| *AO-atn4*-*8*-*12* | *A. oryzae* NSAR1 | pTAex3-*atn4*+*8*+*12* |
| *AO-atn7*-*4*-*8*-*12* | *A. oryzae* NSAR1 | pTAex3-*atn7*+*4*+*8*+*12* |
| *AO-atn1*-*3*-*6*-*9*-*2* | *AO-atn1*-*3*-*6*-*9* | pAdeA-*atn2* |
| *AO-atn1*-*3*-*6*-*9*-*5* | *AO-atn1*-*3*-*6*-*9* | pAdeA-*atn5* |
| *AO-atn1*-*3*-*6*-*9*-*10* | *AO-atn1*-*3*-*6*-*9* | pAdeA-*atn10* |
| *AO-atn1*-*3*-*6*-*9*-*2*-*10* | *AO-atn1*-*3*-*6*-*9* | pAdeA-*atn2+10* |
| *AO-atn1*-*3*-*6*-*9*-*2-5* | *AO-atn1*-*3*-*6*-*9* | pAdeA-*atn2+5* |
| *AO-atn1*-*3*-*6*-*9*-*5*-*10* | *AO-atn1*-*3*-*6*-*9* | pAdeA-*atn5+10* |
| *AO-atn1*-*3*-*6*-*9*-*2*-*10*-*5* | *AO-atn1*-*3*-*6*-*9* | pAdeA-*atn2+10+5* |
| *AO-atn1*-*3*-*6*-*9*-*2*-*10*-*5*-*7* | *AO-atn1*-*3*-*6*-*9* | pAdeA-*atn2+10+5+7* |
| *AO-atn1*-*3*-*6*-*9*-*2*-*10*-*5*-*11* | *AO-atn1*-*3*-*6*-*9* | pAdeA-*atn2+10+5+11* |
| *AO-atn1*-*3*-*6*-*9*-*2*-*10*-*5*-*11-7* | *AO-atn1*-*3*-*6*-*9* | pAdeA-*atn2+10+5+11*+*7* |

**Table S4.** Annotation of each protein in the *atn* cluster. The deduced function of each open reading frame (ORF) and the amino acid sequence similarity/identity, as compared with the homologues found by a BLAST search at NCBI, are shown.

| Gene | Amino acids (base pairs) | Protein homologue (origin) | Similarity/  identity(%) | Proposed function |
| --- | --- | --- | --- | --- |
| *atn1* | 2165 (6498) | SetA (*Aspergillus duricaulis*) | 69/54 | Non-reducing polyketide synthase |
| *atn2* | 294 (885) | AndA (*Aspergillus stellatus*) | 59/39 | Fe(II)/αKG-dependent dioxygenase |
| *atn3* | 307 (1039) | CdmH (*Talaromyces verruculosus*) | 63/40 | UbiA prenyltransferase |
| *atn4* | 512 (1768) | MpaDE (*Penicillium rolfsii*) | 59/38 | Cytochrome P450 |
| *atn5* | 498 (1729) | RAQ58100.1 (*Aspergillus flavus*) | 60/42 | Cytochrome P450 |
| *atn6* | 466 (1595) | CdmI (*Talaromyces verruculosus*) | 65/49 | FAD-dependent monooxygenase |
| *atn7* | 470 (1771) | RDK37504.1 (*Aspergillus phoenicis*) | 62/42 | Cytochrome P450 |
| *atn8* | 301 (1030) | GFF45572.1 (*Aspergillus udagawae*) | 65/49 | NmrA-like reductase |
| *atn9* | 239 (777) | CdmG (*Talaromyces verruculosus*) | 64/48 | Terpene cyclase |
| *atn10* | 260 (963) | CdmF (*Talaromyces verruculosus*) | 70/60 | Short-chain dehydrogenase/reductase |
| *atn11* | 527 (1768) | KAE8162642.1 (*Aspergillus tamarii*) | 64/48 | Cytochrome P450 |
| *atn12* | 157 (474) | BvnE (*Penicillium brevicompactum*) | 56/40 | putative isomerase |

**Table S5. NMR data and structure.**

^1^H (600 MHz) and ^13^C NMR (151 MHz) data for new compound 5 in DMSO-*d_6_*.

| No. | *δ*_H_, (*J* in Hz) | *δ*_C_ | type |
| --- | --- | --- | --- |
| 1 | - | 164.35 | C |
| 2 | - | 102.02 | C |
| 3 | - | 164.76 | C |
| 4 | - | 106.87 | C |
| 5 | - | 155.18 | C |
| 6 | 2.13, s | 17.50 | CH_3_ |
| 7 | 1.85, s | 10.67 | CH_3_ |
| 8 | 3.03, m | 22.51 | CH |
| 9 | 5.07, m | 122.24 | CH |
| 10 | - | 135.16 | C |
| 11 | 1.91, m | 39.69 | CH_2_ |
| 12 | 2.00, m | 26.61 | CH_2_ |
| 13 | 5.08, m | 123.82 | CH |
| 14 | - | 135.53 | C |
| 15 | 1.86, m | 37.13 | CH_2_ |
|  | 2.15, m |  |  |
| 16 | 1.15, m | 29.9 | CH_2_ |
|  | 1.58, m |  |  |
| 17 | 3.02, m | 77.49 | CH |
| 18 | - | 71.55 | C |
| 19 | 1.67, s | 16.53 | CH_3_ |
| 20 | 1.53, s | 16.37 | CH_3_ |
| 21 | 0.97, s | 24.98 | CH_3_ |
| 22 | 1.02, s | 26.71 | CH_3_ |

^1^H (600 MHz) and ^13^C NMR (151 MHz) data for compound **7** in DMSO-*d_6_*.

| No. | *δ*_H_ (*J* in Hz) | *δ*_C_ | type |
| --- | --- | --- | --- |
| 1 | - | 163.2 | C |
| 2 | - | 97.1 | C |
| 3 | - | 162.2 | C |
| 4 | - | 106.1 | C |
| 5 | - | 155.4 | C |
| 6 | 2.14, s | 16.8 | CH_3_ |
| 7 | 1.78, s | 9.2 | CH_3_ |
| 8 | 2.25, dd (16.8, 5.1) | 16.9 | CH_2_ |
| 9 | 1.48, m | 49.7 | CH |
| 10 | - | 79.9 | C |
| 11 | 2.08 m; 1.64 m | 39.1 | CH_2_ |
| 12 | 1.64 m; 1.54 m | 20.0 | CH_2_ |
| 13 | 1.59, m | 53.4 | CH |
| 14 | - | 36.1 | C |
| 15 | 1.85 m; 1.52 m | 37.0 | CH_2_ |
| 16 | 2.59 m; 2.32 m | 33.5 | CH_2_ |
| 17 | - | 215.6 | C |
| 18 | - | 46.6 | C |
| 19 | 1.20, s | 20.2 | CH_3_ |
| 20 | 0.95, s | 14.1 | CH_3_ |
| 21 | 1.03, s | 26.1 | CH_3_ |
| 22 | 0.97, s | 21.0 | CH_3_ |

^1^H (600 MHz) and ^13^C NMR (151 MHz) data for compound **9** in DMSO-*d_6_*.

| No. | *δ*_H_ (*J* in Hz) | *δ*_C_ | type |
| --- | --- | --- | --- |
| 1 | - | 163.1 | C |
| 2 | - | 96.6 | C |
| 3 | - | 162.5 | C |
| 4 | - | 106.4 | C |
| 5 | - | 154.9 | C |
| 6 | 2.15, s | 16.8 | CH_3_ |
| 7 | 1.85, s | 9.3 | CH_3_ |
| 8 | 2.26, m; 2.12, m | 16.2 | CH_2_ |
| 9 | 1.85, m | 42.9 | CH |
| 10 | - | 81.3 | C |
| 11 | 3.89 m | 70.3 | CH |
| 12 | 1.74 m; 1.64 m | 27.1 | CH_2_ |
| 13 | 2.12, m | 45.0 | CH |
| 14 | - | 35.9 | C |
| 15 | 1.56 m; 1.52 m | 37.3 | CH_2_ |
| 16 | 2.55 m; 2.38 m | 33.4 | CH_2_ |
| 17 | - | 215.8 | C |
| 18 | - | 46.0 | C |
| 19 | 1.17, s | 19.7 | CH_3_ |
| 20 | 0.94, s | 13.9 | CH_3_ |
| 21 | 0.96, s | 21.0 | CH_3_ |
| 22 | 0.99, s | 26.1 | CH_3_ |

^1^H (600 MHz) and ^13^C NMR (151 MHz) data for compound **11** in DMSO-*d_6_*.

| No. | *δ*_H_ (*J* in Hz) | *δ*_C_ | type |
| --- | --- | --- | --- |
| 1 | - | 162.8 | C |
| 2 | - | 102.0 | C |
| 3 | - | 162.6 | C |
| 4 | - | 106.6 | C |
| 5 | - | 156.9 | C |
| 6 | 2.17, s | 16.9 | CH_3_ |
| 7 | 1.85, s | 9.2 | CH_3_ |
| 8 | 4.68, dd (10.1, 3.9) | 60.9 | CH |
| 9 | 2.02, d (10.0) | 49.3 | CH |
| 10 | - | 84.0 | C |
| 11 | 3.78, dd (4.0, 2.1) | 70.5 | CH |
| 12 | 1.66, m | 27.0 | CH_2_ |
| 13 | 2.16, m | 45.4 | CH |
| 14 | - | 37.2 | C |
| 15 | 2.34 m; 1.66 m | 39.0 | CH_2_ |
| 16 | 2.48 m; 2.33 m | 33.6 | CH_2_ |
| 17 | - | 216.1 | C |
| 18 | - | 46.5 | C |
| 19 | 1.17, s | 19.8 | CH_3_ |
| 20 | 1.01, s | 14.6 | CH_3_ |
| 21 | 0.98, s | 21.0 | CH_3_ |
| 22 | 0.96, s | 26.2 | CH_3_ |
| 9-OH | 4.75, d (3.9) |  |  |

^1^H (600 MHz) and ^13^C NMR (151 MHz) data for compound **13** in CD_3_OD.

| No. | *δ*_H_ (*J* in Hz) | *δ*_C_ | type |
| --- | --- | --- | --- |
| 1 | - | 167.2 | C |
| 2 | - | 99.0 | C |
| 3 | - | 165.3 | C |
| 4 | - | 108.9 | C |
| 5 | - | 157.2 | C |
| 6 | 2.24, s | 18.0 | CH_3_ |
| 7 | 1.88, s | 9.5 | CH_3_ |
| 8 | 2.21, m | 17.1 | CH_2_ |
| 9 | 2.46, m | 44.3 | CH |
| 10 | - | 81.8 | C |
| 11 | 2.21 m; 1.90 m | 34.7 | CH_2_ |
| 12 | 1.66, m | 27.0 | CH_2_ |
| 13 | 2.16, m | 79.7 | CH |
| 14 | - | 41.9 | C |
| 15 | 2.02 m; 1.68 m | 33.8 | CH_2_ |
| 16 | 2.63 m; 2.48 m | 34.7 | CH_2_ |
| 17 | - | 219.4 | C |
| 18 | - | 54.5 | C |
| 19 | 1.30, s | 20.9 | CH_3_ |
| 20 | 1.17, s | 18.8 | CH_3_ |
| 21 | 1.21, s | 23.9 | CH_3_ |
| 22 | 1.13, s | 22.3 | CH_3_ |

^1^H (600 MHz) and ^13^C NMR (151 MHz) data for compound **16** in CD_3_OD.

| No. | *δ*_H_ (*J* in Hz) | *δ*_C_ | type |
| --- | --- | --- | --- |
| 1 | - | 166.0 | C |
| 2 | - | 103.8 | C |
| 3 | - | 165.4 | C |
| 4 | - | 109.1 | C |
| 5 | - | 159.2 | C |
| 6 | 2.26, s | 17.3 | CH_3_ |
| 7 | 1.95, s | 9.4 | CH_3_ |
| 8 | 4.90, m | 62.5 | CH |
| 9 | 2.72, d (9.8) | 46.3 | CH |
| 10 |  | 85.4 | C |
| 11 | 4.10, t | 74.8 | CH |
| 12 | 2.17 dd (15.2, 2.9); 2.03 dd (15.2, 2.9) | 29.8 | CH_2_ |
| 13 | - | 82.7 | C |
| 14 | - | 44.5 | C |
| 15 | 1.56 m; 1.52 m | 37.3 | CH_2_ |
| 16 | 2.69 m; 2.36 m | 35.0 | CH_2_ |
| 17 | - | 219.4 | C |
| 18 | - | 55.1 | C |
| 19 | 1.30, s | 20.6 | CH_3_ |
| 20 | 1.33, s | 19.7 | CH_3_ |
| 21 | 1.20, s | 24.0 | CH_3_ |
| 22 | 1.20, s | 22.2 | CH_3_ |

^1^H (600 MHz) and ^13^C NMR (151 MHz) data for compound **18** in DMSO-*d_6_*.

| No. | *δ*_H_, mult. (*J* in Hz) | *δ*_C_ | type |
| --- | --- | --- | --- |
| 1 | - | 163.30 | C |
| 2 | - | 102.15 | C |
| 3 | - | 162.70 | C |
| 4 | - | 107.14 | C |
| 5 | - | 157.62 | C |
| 6 | 2.18, s | 17.51 | CH_3_ |
| 7 | 1.85, s | 9.57 | CH_3_ |
| 8 | 4.51, d | 62.70 | CH |
| 9 | 3.49, m | 45.23 | CH |
| 10 | - | 87.24 | C |
| 11 | 3.86, d | 69.76 | CH |
| 12 | 4.37, d | 77.82 | CH |
| 13 | - | 218 | C |
| 14 | 1.47, m | 33.93 | CH |
| 15 | 1.38, m | 34.90 | CH_2_ |
|  | 1.86, m |  |  |
| 16 | 1.42, m | 40.49 | CH_2_ |
|  | 1.90, m |  |  |
| 17 | - | 106.53 | C |
| 18 | - | 51.86 | C |
| 19 | 1.07, s | 15.07 | CH_3_ |
| 20 | 1.18 , d | 19.21 | CH_3_ |
| 21 | 0.91, s | 16.93 | CH_3_ |
| 22 | 0.98, s | 21.56 | CH_3_ |

Supplementary Figures

**Fig. S1.** Analysis of the *Aspergillus versicolor* ADS-F20 fermentation products. (a) HPLC profiles of culture extracts. (b) Chemical structure of compounds **1**, **2**, and **2a**. (c) HRESIMS analysis. (D) UV-visible spectrum analysis.

**Fig. S2.** The HRESIMS spectra of the compounds in the positive ion mode in this study.

**Fig. S3.** The UV-Vis spectra of the compounds in this study.

**Fig. S4.** NMR spectra of compound **3** (DMSO-*d_6_*). (a) ^1^H NMR (600 MHz); (b) ^13^C NMR (151 MHz).

**Fig. S5.** HPLC profiles of mycelial extracts from *A. oryzae* transformants expressing (i) empty vector; (ii) *atn1*+*atn3*.

**Fig. S6.** NMR spectra of compound **5** (DMSO-*d_6_*). (a) ^1^H NMR (600 MHz); (b) ^13^C NMR (151 MHz); (c) ^1^H-^1^H COSY (600 MHz); (d) HSQC (600 MHz); (e) HMBC (600 MHz).

**Fig. S7.** NMR spectra of compound **6** (CD_3_OD). (a) ^1^H NMR (600 MHz); (b) ^13^C NMR (151 MHz); (c) ^1^H-^1^H COSY (600 MHz); (d) HSQC (600 MHz); (e) HMBC (600 MHz).

**Fig. S8.** The SDS-PAGE analysis of purified N-His_6_-tagged recombinant proteins Atn10 (27 kDa) and Atn2 (32.69 kDa). The molecule weights of proteins were calculated based on their protein sequence in Expasy ProtParam tool (https://web.expasy.org/protparam/).

**Fig. S9.** NMR spectra of compound **11** (DMSO-*d_6_*). (a) ^1^H NMR (600 MHz); (b) ^13^C NMR (151 MHz); (c) ^1^H-^1^H COSY (600 MHz); (d) HSQC (600 MHz); (e) HMBC (600 MHz).

**Fig. S10.** The bioconversion analysis of **6** and **9** with *A. oryzae* transformants expressing one of the *atn* genes. The feeding of **6** into the trasnsformants (i) expressing empty vector; (ii) *atn4*+*atn5*+*atn7*+*atn8*+*atn11*+*atn12*; (iv) *atn11*. The feeding of **9** into the trasnsformants expressing (v) *atn4*+*atn5*+*atn7*+*atn8*+*atn11*+*atn12*; (vi) *atn11*. All chromatograms were monitored at 280 nm.

**Fig. S11.** NMR spectra of compound **7** (DMSO-*d_6_*). (a) ^1^H NMR (600 MHz); (b) ^13^C NMR (151 MHz); (c) ^1^H-^1^H COSY (600 MHz); (d) HSQC (600 MHz); (e) HMBC (600 MHz).

**Fig. S12.** NMR spectra of compound **8** (DMSO-*d_6_*). (a) ^1^H NMR (600 MHz); (b) ^13^C NMR (151 MHz); (c) ^1^H-^1^H COSY (600 MHz); (d) HSQC (600 MHz); (e) HMBC (600 MHz).

**Fig. S13.** NMR spectra of compound **12** (DMSO-*d_6_*). (a) ^1^H NMR (600 MHz); (b) ^13^C NMR (151 MHz); (c) ^1^H-^1^H COSY (600 MHz); (d) HSQC (600 MHz); (e) HMBC (600 MHz).

**Fig. S14.** NMR spectra of compound **9** (DMSO-*d_6_*). (a) ^1^H NMR (600 MHz); (b) ^13^C NMR (151 MHz); (c) ^1^H-^1^H COSY (600 MHz); (d) HSQC (600 MHz); (e) HMBC (600 MHz).

**Fig. S15.** NMR spectra of compound **13** (CD_3_OD). (a) ^1^H NMR (600 MHz); (b) ^13^C NMR (151 MHz); (c) ^1^H-^1^H COSY (600 MHz); (d) HSQC (600 MHz); (e) HMBC (600 MHz).

**Fig. S16.** NMR spectra of compound **14** (CD_3_OD). (a) ^1^H NMR (600 MHz); (b) ^13^C NMR (151 MHz); (c) ^1^H-^1^H COSY (600 MHz); (d) HSQC (600 MHz); (e) HMBC (600 MHz).

**Fig. S17.** NMR spectra of compound **10** (DMSO-*d_6_*). (a) ^1^H NMR (600 MHz); (b) ^13^C NMR (151 MHz); (c) ^1^H-^1^H COSY (600 MHz); (d) HSQC (600 MHz); (e) HMBC (600 MHz).

**Fig. S18.** The bioconversion analysis of **10** with *A. oryzae* transformants expressing P450s. Feeding of **10** into the trasnsformant expressing (i) empty vector; (ii) *atn4*; (iii) *atn7*; (v) *atn11.* (iv) The standard of **15**; (vi) The standard of **16**. All chromatograms were monitored at 280 nm.

**Fig. S19.** NMR spectra of compound **15** (DMSO-*d_6_*). (a) ^1^H NMR (600 MHz); (b) ^13^C NMR (151 MHz); (c) ^1^H-^1^H COSY (600 MHz); (d) HSQC (600 MHz); (e) HMBC (600 MHz).

**Fig. S20.** NMR spectra of compound **16** (CD3OD). (a) ^1^H NMR (600 MHz); (b) ^13^C NMR (151 MHz); (c) ^1^H-^1^H COSY (600 MHz); (d) HSQC (600 MHz); (e) HMBC (600MHz).

**Fig. S21.** NMR spectra of compound **18** (DMSO-*d_6_*). (a) ^1^H NMR (600 MHz); (b) ^13^C NMR (151 MHz); (c) ^1^H-^1^H COSY (600 MHz); (d) HSQC (600 MHz); (e) HMBC (600 MHz).

**Fig. S22.** *In vivo* bioconversion analysis between end products **1** and **2**.

**Fig. S23.** NMR spectra of compound **2** (DMSO-*d_6_*). (a) ^1^H NMR (600 MHz); (b) ^13^C NMR (151 MHz); (c) ^1^H-^1^H COSY (600 MHz); (d) HSQC (600 MHz); (e) HMBC (600 MHz).
